# Precision rehabilitation for aphasia by patient age, sex, aphasia severity, and time since stroke? A prespecified, systematic review-based, individual participant data, network, subgroup meta-analysis

**DOI:** 10.1177/17474930221097477

**Published:** 2022-05-18

**Authors:** The RELEASE Collaborators, Marian C Brady, Myzoon Ali, Kathryn VandenBerg, Linda J Williams, Louise R Williams, Masahiro Abo, Frank Becker, Audrey Bowen, Caitlin Brandenburg, Caterina Breitenstein, Stefanie Bruehl, David A Copland, Tamara B Cranfill, Marie di Pietro-Bachmann, Pamela Enderby, Joanne Fillingham, Federica Lucia Galli, Marialuisa Gandolfi, Bertrand Glize, Erin Godecke, Neil Hawkins, Katerina Hilari, Jacqueline Hinckley, Simon Horton, David Howard, Petra Jaecks, Elizabeth Jefferies, Luis MT Jesus, Maria Kambanaros, Eun Kyoung Kang, Eman M Khedr, Anthony Pak-Hin Kong, Tarja Kukkonen, Marina Laganaro, Matthew A Lambon Ralph, Ann Charlotte Laska, Béatrice Leemann, Alexander P Leff, Roxele R Lima, Antje Lorenz, Brian MacWhinney, Rebecca Shisler Marshall, Flavia Mattioli, İlknur Maviş, Marcus Meinzer, Reza Nilipour, Enrique Noé, Nam-Jong Paik, Rebecca Palmer, Ilias Papathanasiou, Brigida Patricio, Isabel Pavão Martins, Cathy Price, Tatjana Prizl Jakovac, Elizabeth Rochon, Miranda L Rose, Charlotte Rosso, Ilona Rubi-Fessen, Marina B Ruiter, Claerwen Snell, Benjamin Stahl, Jerzy P Szaflarski, Shirley A Thomas, Mieke van de Sandt-Koenderman, Ineke van der Meulen, Evy Visch-Brink, Linda Worrall, Heather Harris Wright

**Keywords:** Stroke, aphasia, rehabilitation, speech and language therapy, individual participant data, network meta-analysis

## Abstract

**Background:**

Stroke rehabilitation interventions are routinely personalized to address individuals’ needs, goals, and challenges based on evidence from aggregated randomized controlled trials (RCT) data and meta-syntheses. Individual participant data (IPD) meta-analyses may better inform the development of precision rehabilitation approaches, quantifying treatment responses while adjusting for confounders and reducing ecological bias.

**Aim:**

We explored associations between speech and language therapy (SLT) interventions frequency (days/week), intensity (h/week), and dosage (total SLT-hours) and language outcomes for different age, sex, aphasia severity, and chronicity subgroups by undertaking prespecified subgroup network meta-analyses of the RELEASE database.

**Methods:**

MEDLINE, EMBASE, and trial registrations were systematically searched (inception-Sept2015) for RCTs, including ⩾ 10 IPD on stroke-related aphasia. We extracted demographic, stroke, aphasia, SLT, and risk of bias data. Overall-language ability, auditory comprehension, and functional communication outcomes were standardized. A one-stage, random effects, network meta-analysis approach filtered IPD into a single optimal model, examining SLT regimen and language recovery from baseline to first post-intervention follow-up, adjusting for covariates identified *a-priori*. Data were dichotomized by age (⩽/> 65 years), aphasia severity (mild–moderate/ moderate–severe based on language outcomes’ median value), chronicity (⩽/> 3 months), and sex subgroups. We reported estimates of means and 95% confidence intervals. Where relative variance was high (> 50%), results were reported for completeness.

**Results:**

959 IPD (25 RCTs) were analyzed. For working-age participants, greatest language gains from baseline occurred alongside moderate to high-intensity SLT (functional communication 3-to-4 h/week; overall-language and comprehension > 9 h/week); older participants’ greatest gains occurred alongside low-intensity SLT (⩽ 2 h/week) except for auditory comprehension (> 9 h/week). For both age-groups, SLT-frequency and dosage associated with best language gains were similar. Participants ⩽ 3 months post-onset demonstrated greatest overall-language gains for SLT at low intensity/moderate dosage (⩽ 2 SLT-h/week; 20-to-50 h); for those > 3 months, post-stroke greatest gains were associated with moderate-intensity/high-dosage SLT (3–4 SLT-h/week; ⩾ 50 hours). For moderate–severe participants, 4 SLT-days/week conferred the greatest language gains across outcomes, with auditory comprehension gains only observed for ⩾ 4 SLT-days/week; mild–moderate participants’ greatest functional communication gains were associated with similar frequency (⩾ 4 SLT-days/week) and greatest overall-language gains with higher frequency SLT (⩾ 6 days/weekly). Males’ greatest gains were associated with SLT of moderate (functional communication; 3-to-4 h/weekly) or high intensity (overall-language and auditory comprehension; (> 9 h/weekly) compared to females for whom the greatest gains were associated with lower-intensity SLT (< 2 SLT-h/weekly). Consistencies across subgroups were also evident; greatest overall-language gains were associated with 20-to-50 SLT-h in total; auditory comprehension gains were generally observed when SLT > 9 h over ⩾ 4 days/week.

**Conclusions:**

We observed a treatment response in most subgroups’ overall-language, auditory comprehension, and functional communication language gains. For some, the maximum treatment response varied in association with different SLT-frequency, intensity, and dosage. Where differences were observed, working-aged, chronic, mild–moderate, and male subgroups experienced their greatest language gains alongside high-frequency/intensity SLT. In contrast, older, moderate–severely impaired, and female subgroups within 3 months of aphasia onset made their greatest gains for lower-intensity SLT. The acceptability, clinical, and cost effectiveness of precision aphasia rehabilitation approaches based on age, sex, aphasia severity, and chronicity should be evaluated in future clinical RCTs.

## Introduction

Personalized health care is a central tenet of stroke rehabilitation; therapists routinely tailor interventions to patients’ individual goals, preferences, the optimal difficulty level, the local environment, risks, their health, and cognitive status and limitations.^1^ Precision medicine refers to data-driven decision making based on patient subpopulations and responsiveness to specific interventions.^2^ Data-driven “precision” rehabilitation intervention decisions, with gains in acceptability, clinical, and cost effectiveness, require exploration of subgroups’ responsiveness to interventions based on, for example, clinical demographics, stroke severity, and time post-onset.^3^

### Effectiveness of rehabilitation interventions

Examining the effectiveness of complex rehabilitation interventions is challenging.^4^ Aphasia interventions, for example, vary by regime (frequency, intensity, duration, and dosage), provider, delivery model, and therapeutic approach. Therapists’ clinical decision making is typically informed by findings from high-quality, group-level comparisons within randomized controlled trials (RCTs) and estimates of the average effect based on meta-synthesis of aggregate group data, which in turn inform clinical guidelines.^5,6^ High-level aphasia rehabilitation evidence has, to date, been primarily based on aggregate data from high-quality RCTs and meta-synthesis,^5,7^ thus limiting the degree to which it might inform precision rehabilitation decisions.^8^

### Aggregate data limitations

Aggregate data based on relatively large samples and adequate statistical power provide crucial evidence of the effectiveness of rehabilitation interventions across recruited populations. The ability of an isolated trial to determine subgroups’ differential treatment response, however, is limited.^8,9^ Limitations to aggregate data meta-syntheses include the risk of outcome reporting bias,^10^ restricted inclusion of language data, and the need to synthesize various clinical language assessments into a standardized mean difference^11,12^, making clinical interpretation and application of emerging evidence a challenge.^9,13^ In addition, meta-syntheses of aggregate data risk masking the responsiveness of different subgroups to specific interventions, known as ecological bias.^14^ Aphasia meta-syntheses have presented findings of interest (and concern); for example, one meta-analysis highlighted the benefits of intensive SLT early post-onset though these findings were confounded by higher participant dropouts (n = 35/114) compared to lower-intensity SLT participants (n = 17/102; p = 0.01).^5^ In contrast, among the RCTs which recruited participants years post-onset, neither dropouts nor significant language gains were evident.^5^ Further examination of the potential benefits, risks, and evident variability in tolerance and responsiveness to the different SLT regimens may be worthwhile but could not be advanced until individual participants’ data (IPD) on demographics were available.

### Individual participant data meta-analyses

Where strong theoretical reasons and clinical plausibility suggest differential responses to an intervention (e.g., aphasia rehabilitation), large IPD meta-analysis is the gold standard.^14^ Our earlier IPD meta-analysis found that trial participants’ greatest overall and auditory comprehension language gains were associated with > 20–50 h SLT dosage, delivered 2-to-4 h each week, and between 3 and 5 + days. Greatest auditory comprehension gains were associated with 9 + SLT-h weekly over 4-to-5 days.^15^ Meta-analyses using IPD also support the exploration of differential treatment response across participants,^8,16^ reducing the risk of ecological bias, facilitating the inclusion of previously unreported dropouts, outcome measurement, and follow-up data while increasing statistical power.^9,10,17^ In a highly heterogeneous population such as people with aphasia, investigations of subgroups’ (based on age, aphasia severity, chronicity, or sex) treatment responsiveness might be explored with greater statistical power and adjustments for confounders than at trial level.^9,14,18^ Information on differential responsiveness of clinically relevant subgroups may inform the development of precision aphasia rehabilitation approaches and future RCT-based treatment evaluations

## Aims

Following a large IPD network meta-analysis,^15^ our prespecified subgroup analysis aimed to explore language change from baseline to first post-intervention follow-up (measuring overall-language, auditory comprehension, and functional communication) for various levels of rehabilitation, intensity, dosage in aphasia subgroups, varying by participants’ age, sex, aphasia chronicity and severity at baseline.

## Methods

Approval was granted for this database study (UK IRAS registration ID 179505; Glasgow Caledonian University Health and Life Sciences Ethical Committee HLS/NCH/15/09).

### Search strategy and selection criteria

We created the international RELEASE IPD database of aphasia research to support several planned analyses,^15,19,20^ systematically identifying and reviewing published and unpublished datasets with ⩾ 10 IPD on aphasia, language outcome, and time since stroke (reported in full elsewhere).^15,19,21^ Briefly, several electronic databases, including MEDLINE and EMBASE, were searched (inception-September 2015 for eligible datasets). Anticipating lengthy data cleaning and analysis procedures, we also searched trial registrations for eligible trials completing beyond the electronic search date.^15,21^ Non-English datasets were translated. Language data derived from stroke or screening measures were excluded. Non-randomized trials, case series, and clinical registries were included in the database and supported previous analyses^20^ but were excluded from this subgroup analysis.

Full text reports were reviewed independently by two reviewers; a third resolved disagreements. Potentially eligible datasets were invited to contribute IPD. One reminder was sent to non-respondents, followed by attempts to contact co-authors. We confirmed dataset eligibility with respondents prior to contribution. A protocol guided data searching, identification, extraction, and analyses (PROSPERO CRD42018110947).^21^ Included datasets reported relevant ethical and gatekeeper approvals.

### Data extraction and preparation

For this prespecified subgroup analysis, we extracted data on demography (including sex, age, and language used), stroke (time post-onset, hemisphere, and aphasia severity), SLT intervention, and language outcome (overall-language ability, auditory comprehension, and functional communication). Language recovery was defined as the change in absolute language score from baseline to first post-intervention follow-up. We checked and collated language domain measurements, as agreed *a priori* by the RELEASE collaborators. Whenever possible, baseline and subsequent timepoint data extraction was confirmed and additional unreported data sought from the primary researchers.^21^

For each outcome, the most frequently used measure by dataset was identified as the *anchor measure.* All remaining measures of that outcome *(minority measures)* were transformed to match the anchor measure’s range and format, thus retaining the clinical relevance of the anchor measures’ change score.^19^ Anchor measures comprised the Western aphasia battery-aphasia quotient (WAB-AQ)^22^ for overall-language ability; the Aachen Aphasia test-token test (AAT-TT) scored positively for auditory comprehension;^23^ and the Aachen Aphasia test-spontaneous speech communication (AAT-SSC) rating domain score for functional communication.^23^

SLT interventions targeting language recovery were categorized by regimen (frequency, intensity, and dosage; Supplemental Material A). All data were checked by an independent researcher and, where possible, with available documentation and primary researchers. We recorded unavailable data as “*unreported*.” Aphasia with a non-stroke etiology, unreported time post-onset, and duplicate IPD were excluded. In the absence of SLT intervention records at IPD level, we applied group-level SLT descriptions to the IPD accordingly. Final data formatting decisions were made through collaborator discussion. Categorical data formats (e.g., 5-to-6 weeks) were recorded as means (5.5 weeks). Language co-interventions (e.g., pharmacological) were documented and IPD were included up to the point of any crossover.

### IPD network meta-analysis

Drawing only RCT IPD from the wider RELEASE database (21), we conducted a network meta-analysis of SLT interventions delivered by language outcomes (15). Where traditional meta-analyses consider pair-wise trial comparisons (e.g., treatment 1 vs 2), network meta-analysis considers three or more interventions simultaneously by making direct (treatment 1 vs 2; treatment 2 vs 3) and indirect comparisons (treatment 1 vs 3), thus yielding more precise estimates than paired direct/indirect estimates and making it possible to compare the effectiveness of interventions. We used datasets as random effects and demographics and interventions as fixed effects. Data analysis for this article used SAS™ software (9.4 using PROCMIXED). Using a statistical inferencing approach, we sought to highlight important research questions, considerations for future trial design, and clinical implementation. Language recovery was defined as the mean of the absolute change from baseline to the first follow-up on the transformed standardized measure. Effect sizes were estimated and reported (95% CI). Our minimum sample size for each analysis was 20 IPD (2 RCTs).

We included prespecified potential confounders in the base model (age, sex, aphasia severity, and time post-onset) and simultaneously examined the impact of IPD and language variables on the intervention effect. Our one-stage network meta-analysis examined IPD and SLT intervention regimen variations by age, time since onset, aphasia severity at baseline and sex subgroups, and associated estimates of mean language gains from baseline (Supplemental Material A). Continuous regimen variables were grouped for comparison (e.g., 3 vs 4 SLT-days/week). We dichotomized IPD based on key demographic and clinical data; males and females, median age (working age ⩽ 65 years and older > 65 years; rehabilitation timing after aphasia onset (early ⩽ 3 months and late > 3 months); moderate–severe and mild–moderate groups based on the overall median language modality score.

We considered clustering by dataset distinguishing IPD from dataset-based interactions.^15,21^ Where > 20% of a dataset variable was missing, we excluded it from that network analysis. Patterns of loss were checked; we compared missing data to demographic and other variables using the t-test or Mann–Whitney *U* test. In the absence of evidence of influence, we studied the data missing at random. We excluded any data not missing at random. For risk of bias and heterogeneity checking, see Supplemental Material B.

## Results

Filtering by available demographic, language, intervention data, and time points, 959 IPD (25 RCTs; Supplemental Material C to F) informed a prespecified IPD subgroup network meta-analysis of therapy regimen and language outcomes: overall-language ability (WAB-AQ 482 IPD; 11 RCTs); functional communication (AAT-SSC 533 IPD; 14 RCTs); and auditory comprehension (AAT-TT 550 IPD; 16 RCTs). Participants experienced predominantly left hemisphere (683 IPD; 97.7%); ischemic first strokes (685 IPD; 88.9%) with English predominant across languages represented (255 IPD; 26.6%; Supplemental Material G). We examined within study clustering; the findings were non-significant or caused a model failure (the G-matrix was not positive definite).^20,21^ Network geometries were stable.^15^ Overall, the greatest language gains from baseline to the first follow-up occurred among working-age, female, moderate–severe aphasia severity subgroups, and those within 3 months of stroke onset (Table 1; Supplemental Material H to K).

**Table 1.** Subgroup analysis; greatest significant gains [95% CI] from baseline by SLT regimen, language outcome, and IPD (RCTs).

Subgroup	Frequency* days weekly	*Points [95% CI]* *IPD (RCTs)*	Intensity* hours weekly	*Points [95% CI]* *IPD (RCTs)*	Dosage* total SLT-hours	*Points [95% CI]* *IPD (RCTs)*
Overall-language ability: WAB-AQ 0–100 points						
⩽ 65 years	5 *(⩾* *3-to-5)*	15.1 [8.2, 22.1] IPD 107 (6)	> 9 *(<* *2)*	17.0 [10.0, 24.0] IPD 78 (3)	20-to-50 *(>* *50)*	23.4 [13.5, 33.3] IPD 15(3)
> 65 years	⩾ 5 *(4)*	17.2 [3.9, 30.5] IPD 10 (2)	up to 2 *(3-to-4)*	16.9 [3.8, 30.0] IPD 37 (3)	20-to-50 *(14-to-20)*	15.95 [5.1, 26.8] IPD 16(4)
SLT ⩽ 3 months	5 *(⩾* *4)*	27.7 [3.6, 51.9] IPD 3 (1)	up to 2 *(3-to-4 and* *>* *9)*	24.3 [13.4, 35.2] IPD 62 (2)	20-to-50	27.5 [18.3, 36.7] IPD 27(3)
SLT > 3 months	5 *(⩾* *4)*	6.32 [1.6, 11.1] IPD 44 (1)	3-to-4 *(>* *9)*	6.3 [2.2, 10.3] IPD 25 (2)	⩾ 50	10.1 [4.2, 16.0] IPD 15 (1)
Severe–moderate aphasia	4 *(2-to* *>* *5)*	18.3 [8.0, 28.5] IPD 38 (5)	3-to-4 *(up to 2 and* *>* *9)*	20.0 [10.4, 28.8] IPD 48 (3)	20-to-50 *(5-to-50)*	23.5 [13.5, 33.5] IPD 23 (3)
Mild–moderate aphasia	> 5 *(2-to* *>* *5)*	9.9 [4.4, 15.3] IPD 16 (2)	> 9 (up to 2 and > 3)	8.0 [3.4, 12.6] IPD 48 (3)	20-to-50 *(5-to-50)*	8.7 [2.0, 15.5] IPD 8 (2)
Female	5 *(2-to* *>* *5)*	24.2 [7.7, 40.7] IPD 5 (2)	up to 2 *(2 to* *>* *9)*	18.5 [7.5, 29.6] IPD 37 (3)	20-to-50	24.4 [12.2, 36.5] IPD 13 (2)
Male	⩾ 5 *(⩾* *4)*	13.6 [4.3, 22.9] IPD 21 (2)	> 9 *(up to 2 and 3-to-4)*	15.1 [7.9, 22.3] IPD 61 (3)	20-to-50 *(2-to-20)*	15.6 [6.2, 25.0] IPD 18 (4)
Auditory comprehension: AAT-TT 0–50 points						
⩽ 65 years	4 *(5)*	6.8 [2.3, 11.2] IPD 64 (5)	> 9	9.0 [5.4, 12.6] IPD 108 (4)	20-to-50	6.1 [1.8, 10.4] IPD 59(6)
> 65 years	4	8.5 [2.0, 14.9] IPD 50 (3)	> 9	5.3 [0.7, 10.0] IPD 33(6)	20-to-50	5.8 [1.3, 10.2] IPD 34(7)
SLT ⩽ 3 months	NS	NS	> 9	9.3 [2.1, 16.5] IPD 20 (2)	NS	NS
SLT > 3 months	5 *(4)*	3.7 [1.4, 6.0] IPD 89 (5)	> 9	4.6 [2.4, 6.8] IPD 121 (4)	⩾ 50 *(>* *14-to-50)*	4.2 [1.7, 6.7] IPD 76 (3)
Severe–moderate aphasia	4	8.5 [3.7, 13.3] IPD 80 (5)	> 9 *(up to 2)*	9.1 [2.6, 15.6] IPD 9 (2)	⩾ 50 *(>* *14-to-20)*	8.9 [4.5, 13.3] IPD 142 (6)
Mild–moderate Aphasia	NS	NS	NS	NS	NS	NS
Female	4 *(5)*	8.1 [2.7, 13.6] IPD 52 (5)	up to 2 *(3-to-4 and* *>* *9)*	7.9 [1.8, 14.0] IPD 10 (2)	⩾ 50 *(20-to-50)*	7.0 [2.9, 11.2] IPD 77 (6)
Male	5 *(4)*	5.1 [1.4, 8.7] IPD 99 (8)	> 9 *(3-to-4)*	7.6 [3.8, 11.5] IPD 91 (6)	20-to-50	6.5 [1.8, 11.2] IPD 56 (7)
Functional communication: AAT-SSC, score 0–5 points						
⩽ 65 years	⩾ 5	1.2 [0.3, 2.1] IPD 3 (2)	3-to-4	0.9 [0.5, 1.2] IPD 74 (5)	14-to-20 *(⩾* *50)*	1.1 [0.5, 1.8] IPD 6 (3)
> 65 years	4 *(5)*	0.8 [0.2, 1.4] IPD 54 (3)	up to 2	1.0 [0.4, 1.6] IPD 41 (4)	20-to-50 *(>* *5-to-14)*	0.86 [0.4, 1.4] IPD 31(9)
SLT ⩽ 3 months	4	1.6 [ 0.6, 2.5] IPD 80 (1)	up to 3	1.3 [0.6, 1.9] IPD 57 (2)	20-to-50	1.2 [0.7, 1.8] IPD 31 (3)
SLT > 3 months	NS	NS	NS	NS	NS	NS
Severe–moderate aphasia	4	1.0 [0.5, 1.5] IPD 55 (2)	< 2-to-3	1.0 [0.3, 1.6] IPD 42 (3)	⩾ 50 *(14-to-20)*	1.1 [0.4, 1.8] IPD 9 (3)
Mild–moderate aphasia	> 5 *(⩾* *4)*	0.7 [0.4, 1.0] IPD 55 (8)	> 9 *(<* *2 and* *>* *4)*	0.63 [0.26, 1.0] IPD 27 (4)	> 14-to-20 *(⩾* *50)*	0.7 [0.2, 1.2] IPD 8 (3)
Female	4 *(3)*	1.0 [0.2, 1.8] IPD 49(3)	up to 2	1.2 [0.5, 1.8] IPD 39(4)	> 14-to-20 *(5-to-⩾* *50)*	1.6 [0.6, 2.6] IPD 4 (2)
Male	> 5	0.8 [0.1, 1.5] IPD 6(3)	3-to-4 *(>* *9)*	0.7 [0.4, 0.9] IPD 97(5)	⩾ 50 *(20-to-50)*	0.7 [0.5, 0.9] IPD 93 (6)

Key: MD mean difference. Underline text > 50% relative covariance reported for completeness. Bold refers to greatest gain associations. *Italics refer to clinically similar gains.*

### Age and language rehabilitation

#### Frequency

Working-age participants’ greatest overall-language gains occurred for 5 SLT-days/week (similar gains observed for 3-to-6 + days/week) and for older participants’ ⩾ 6 SLT-days/week (10 IPD; followed by 4 SLT-days/week; 28 IPD). Auditory comprehension gains were absent when SLT < 4 days/week. The greatest gains for both age-groups were observed at 4 SLT-days/week, the only significant gain for older participants; working-aged participants made similar gains at 5 days/week. Working-age participants’ greatest functional communication gains occurred for ⩾ 5 SLT-days/week and were observed for older participants for 4 SLT-days/week (with similar gains at 5 SLT-days/week; Table 1; Supplemental Material H(a) to (c)).

#### Intensity

Working-age participants made their greatest overall-language and auditory comprehension gains alongside > 9 SLT-h/week. Older participants’ greatest overall-language gains occurred for < 2 SLT-h/week (similar for 3–4 SLT-h/week) while their only comprehension gain occurred when SLT > 9 h/week. Functional communication gains were greatest for working-age participants’ when SLT 3–4 h/week and ⩽ 2 h/week for the older (Table 1; Supplemental Material H(d) to (f)).

#### Dosage

Both age-groups’ greatest overall-language gains occurred alongside 20-to-50 SLT-h (few IPD; see Table 1) while other gains, based on greater IPD, were similar across dosages. Working-age participants’ auditory comprehension gains were greatest for 20–50 SLT-h (with similar gains for 14–20 h); older participants’ significant gains were only observed for > 20 SLT-h. Older participants made greatest functional communication gains alongside 20–50 SLT-h; for working-age participants gains observed for 14-to-20 SLT-h were based on 6 IPD, followed by > 50 SLT-h (87 IPD) (Table 1; Supplemental Material H(g) to (i)).

### Early versus late rehabilitation

#### Frequency

The greatest overall-language gains were observed for three participants that received early SLT 3 days/week, but the greatest gains for most early-rehabilitation participants (150 IPD) were noted for 5 SLT-days/week. Significant auditory comprehension gains were not observed, but functional communication gains were greatest for early 4 SLT-days/week. Participants that received SLT > 3 months post-aphasia onset in a trial context made greatest overall-language and auditory comprehension gains for 5 SLT-days/week. No significant functional communication gains from baseline were observed in this late-rehabilitation group (Table 1; Supplemental Material I(a) to (c)).

#### Intensity

The early-SLT group’s greatest overall-language gains occurred for up to 2 SLT-h/week with similar gains observed for 3-to-4 and > 9 SLT-h/week. Overall-language gains for late-rehabilitation participants were less pronounced, with the greatest of these associated with SLT 3-to-4 h/week, with similar gains when SLT > 9 h/week. Auditory comprehension gains were only observed when SLT > 9 h/week regardless of the timing of the intervention. No functional communication gains were observed for the late-SLT group; the early-SLT group’s gains were greatest for 2–3 SLT-h/week (Table 1; Supplemental Material I(d) to (f)).

#### Dosage

The early-SLT group achieved their greatest overall-language and functional communication gains for 20–50 SLT-h; auditory comprehension gains were not observed at any dosage. The late-SLT group’s only significant overall-language gain from baseline occurred for > 50 SLT-h; gains in auditory comprehension were significant for > 14 SLT-h with the greatest of these for > 50 SLT-h. The late-SLT group made no significant functional communication gains from baseline at any dosage (Table 1; Supplemental Material I(g) to (i)).

### Aphasia severity and language rehabilitation

#### Frequency

When SLT was 4 days/week, participants with moderate–severe aphasia experienced the greatest overall-language, auditory comprehension, and functional communication gains. Comprehension gains were only observed for ⩾ 4 SLT-days/week. In contrast, the mild–moderate group’s greatest gains occurred for ⩾ 6 SLT-days/week (overall language) and functional communication from ⩾ 4 SLT-days/week. Relative variance for auditory comprehension analysis was high (> 50%) (Table 1; Supplemental Material J(a) to (c)).

#### Intensity

The moderate–severe group made significant gains on overall language at all intensities, with greatest gains for 3-to-4 SLT-h/week. Their greatest auditory comprehension gains were observed for < 2 SLT-h/week but based on few participants, while similar gains were observed for > 9 SLT-h/week but informed by more IPD.

When SLT was > 9 h/week, the mild–moderate group made their greatest overall-language gains (similar gains < 2 and > 3 SLT-h/week). They made significant functional communication gains across intensities. Relative variance of the auditory comprehension analysis for this group was > 50% (Table 1; Supplemental Material J(d) to (f)).

#### Dosage

The moderate–severe group made greatest overall-language gains for 20-to-50 SLT-h with similar gains for 5–50 + SLT-h. Greatest gains were observed for the mild–moderate group for > 20 h. The relative variance was > 50% for the mild–moderate group’s auditory comprehension analysis; no gains were observed. The moderate–severe group’s greatest comprehension and functional communication gains occurred for ⩾ 50 SLT-h. The mild–moderate group made significant functional communication gains at all dosages (the greatest observed for > 14–20 SLT-h but based on 8 IPD). (Table 1; Supplemental Material J(g) to (i)).

### Sex and language rehabilitation

#### Frequency

Females’ greatest overall-language gains occurred for 3 SLT-days/week (5 IPD) followed by 5 SLT-days/week; similar gains observed across frequencies. Males’ greatest overall-language gains were for ⩾ 6 SLT-days/week (similar gains for 4-to-5 SLT-days/week) while greatest functional communication gains occurred for ⩾ 5 SLT-day/week. Females’ functional communication gains were greatest for 4 SLT-days/week with similar gains observed for 3 SLT-days/week. Both female and male groups’ greatest and only comprehension gains occurred for 4-to-5 SLT-days/week. No auditory comprehension gains were observed when SLT < 4 or 6 days/week (Table 1; Supplemental Material K(a) to (c)).

#### Intensity

Females’ greatest overall-language and functional communication gains occurred for < 2 SLT-h/week as did their greatest auditory comprehension gains (the latter based on few IPD). The next greatest comprehension gains were observed for ⩾ 9 SLT-h/weekly. Similar overall-language gains also occurred at 2 to > 9 SLT-h/week.

Males’ greatest overall-language and comprehension gains occurred for > 9 SLT-h/week, but greatest functional communication gains occurred for 3–4 SLT-h/week. Similar gains were observed for > 9 SLT-h/week for overall language, auditory comprehension. Comprehension gains among male participants were absent when SLT ⩽ 3 h/week (Table 1; Supplemental Material K(d) to (f)).

#### Dosage

Females and males’ greatest overall-language gains occurred alongside 20-to-50 SLT-h (males made similar gains for > 50 SLT-h). For males’ greatest comprehension, gains were also observed for 20-to-50 SLT-h, whereas females’ comprehension gains were greatest for > 50 SLT-h. Comprehension gains were absent for both groups for < 20 SLT-h. Females’ greatest functional communication gains occurred for > 14–20 SLT-h (based on few IPD; gains observed at all dosages > 5 SLT-h). Males’ functional communication was greatest alongside ⩾ 50 SLT-h with clinically similar gains observed for > 20–50 SLT-h (Table 1; Supplemental Material K(g) to (i)).

## Discussion

Our IPD network meta-analysis of 959 individual datasets (25 RCTs) explored patterns of interaction between SLT-frequency, intensity, and dosage and aphasia language outcomes by age, sex, aphasia chronicity and severity subgroups. Some subgroup consistencies were evident; greatest overall-language gains were associated with 20–50 SLT-h; comprehension gains were only evident > 9 SLT-h over ⩾ 4 SLT-days/week.^15^ Most subgroups demonstrated gains from baseline to first post-intervention follow-up across outcomes, consistent with previous pairwise, aggregate data, and meta-analyses.^5,7^ Our findings also suggest that differential aphasia rehabilitation responses may exist for some subgroups. Older participants’ overall-language gains were greatest when associated with lower-intensity SLT than working-age participants’ gains (⩽ 2 vs > 9 SLT-h/week); optimal frequency and dosage were similar across groups. Generally, early-intervention participants’ greatest overall-language gains occurred for up to 2 SLT-h/week for 20-to-50 h; when SLT > 3 months post-onset greatest gains occurred for 3-to-4 SLT-h/week for ⩾ 50 h. Moderate–severe participants made significant overall-language gains across intensities (greatest for 3–4 SLT-h/weekly); functional communication gains were greatest for < 3 SLT-h/week. In contrast, mild–moderate participants’ gains were associated with higher-intensity SLT (> 9 h/week). Males’ greatest language gains were associated with high-frequency and -intensity SLT (⩾ 5 SLT-days/week; > 9 h/week), while females’ greatest language gains were associated with SLT-frequency and intensity of 4-to-5 SLT-days/week; < 2 h/weekly.

Previous aggregate meta-analyses suggested optimal SLT intensities were ⩾ 2 SLT-h/week^24^ or in the region of 9 SLT-h/week^7^, while dosage of > 90 SLT-h was likely to confer language benefit but < 44 SLT-h may not.^24^ Our analysis refines these estimates, highlighting variations by language outcome and subgroups.

Subgroup meta-analyses carry intrinsic strengths and limitations.^25,26^ The high number of diverse IPD (including public domain datasets, languages, clinical, and regional contexts) sought to maximize data inclusion, ensure sufficient data overlap, and support generalization of findings. Our inclusion of trials completed beyond our search–end date ensured the dataset’s currency. Trial registration is a requirement met by high-quality trials, but an unregistered trial may have been missed from this search strategy. Strong rationale, early empirical evidence, and plausible clinical perceptions of differential responsiveness to SLT supported our multiple, planned, and subgroup network meta-analyses. Included RCTs had a low risk of bias (Supplemental Material N), and of the 25 included RCTs, only ten participants did not have the data points necessary to contribute to our planned analysis. Our meta-synthesis preserved clinically relevant measurements, supporting clinical interpretation.

We acknowledge that spontaneous recovery may also impact on treatment gains observed, with median overall-language gains higher for the early-rehabilitation subgroup than the late-rehabilitation group. In addition, patients in the acute stroke stage may have reduced capacity to engage in SLT or RCT activities.^6^ The extent of such impacts remains to be determined. Our exploratory IPD meta-analysis reflects highly selected participants, interventions, language outcomes, and the availability of sufficiently detailed records within included RCTs. Where limited data were available, there remain uncertainties in our findings. Language gains observed reflect the change from RCT baseline to the first follow-up only. Pre-randomization SLT and language change data were unavailable. Concurrent impairment or comorbidities were rarely reported. Other demographic data were inconsistently available.^19^ Our analysis controlled for time post-stroke and aphasia severity, though other threats to the validity of the effect estimation may exist, including participants’ tolerance to highly intensive SLT early post-stroke. Our statistical inferencing, hypothesis generating approach carries a risk of false negative and positive findings. Replication of these findings through confirmatory clinical trials is required.

### Clinical implications

Greatest overall-language and auditory comprehension gains across subgroups were associated with higher dosage levels > 20–50 SLT-h or above) than current clinical provision reported for 4–16 h.^27–30^ Higher dosage rehabilitation within existing resources may be challenging, requiring alternative delivery models to achieve the requisite dose, such as telerehabilitation, self-management, trained family members, prescribed home-practice tasks, and group therapy. Our findings also highlighted frequency–intensity–dosage ranges below which language gains from baseline were not observed. Some plausible, clinically relevant subgroups may benefit from precision rehabilitation approaches based on age, aphasia severity, chronicity, and sex.

### Research implications

High-quality targeted SLT RCTs should be conducted to evaluate the acceptability, clinical, and cost effectiveness of precision aphasia rehabilitation approaches. Participants should be selected by age-group, time since stroke, and severity or stratifying intervention by subgroup. Despite no language restrictions, our predominantly English-speaking participant data highlighted an underrepresentation of non-English aphasia research. Minimal SLT-frequency–intensity–dosage levels should be applied to intervention development and evaluation, in addition to stratification by age, time post-stroke, and severity. Continued collaborative approaches including research data sharing will support the reduction of research waste and further insights into precision stroke rehabilitation, including SLT for aphasia.

## Conclusion

Exploratory IPD meta-analysis based on aphasia RCT IPD demonstrated that most subgroups with aphasia made significant overall-language ability, auditory comprehension, and functional communication gains from baseline and suggested that some subgroups may achieve their greatest language gains in the context of specific SLT-frequency, intensity, and dosage regimens. Where differences arose, older, moderate–severely impaired, and female subgroups’ greatest gains were associated with lower-intensity SLT. Working-aged, mild–moderate aphasia, and male subgroups’ greatest language gains were associated with high-frequency/intensity SLT.

## Supplemental Material

sj-docx-1-wso-10.1177_17474930221097477 – Supplemental material for Precision rehabilitation for aphasia by patient age, sex, aphasia severity, and time since stroke? A prespecified, systematic review-based, individual participant data, network, subgroup meta-analysis Click here for additional data file. Supplemental material, sj-docx-1-wso-10.1177_17474930221097477 for Precision rehabilitation for aphasia by patient age, sex, aphasia severity, and time since stroke? A prespecified, systematic review-based, individual participant data, network, subgroup meta-analysis by Marian C Brady, Myzoon Ali, Kathryn VandenBerg, Linda J Williams, Louise R Williams, Masahiro Abo, Frank Becker, Audrey Bowen, Caitlin Brandenburg, Caterina Breitenstein, Stefanie Bruehl, David A Copland, Tamara B Cranfill, Marie di Pietro-Bachmann, Pamela Enderby, Joanne Fillingham, Federica Lucia Galli, Marialuisa Gandolfi, Bertrand Glize, Erin Godecke, Neil Hawkins, Katerina Hilari, Jacqueline Hinckley, Simon Horton, David Howard, Petra Jaecks, Elizabeth Jefferies, Luis MT Jesus, Maria Kambanaros, Eun Kyoung Kang, Eman M Khedr, Anthony Pak-Hin Kong, Tarja Kukkonen, Marina Laganaro, Matthew A Lambon Ralph, Ann Charlotte Laska, Béatrice Leemann, Alexander P Leff, Roxele R Lima, Antje Lorenz, Brian MacWhinney, Rebecca Shisler Marshall, Flavia Mattioli, İlknur Maviş, Marcus Meinzer, Reza Nilipour, Enrique Noé, Nam-Jong Paik, Rebecca Palmer, Ilias Papathanasiou, Brigida Patricio, Isabel Pavão Martins, Cathy Price, Tatjana Prizl Jakovac, Elizabeth Rochon, Miranda L Rose, Charlotte Rosso, Ilona Rubi-Fessen, Marina B Ruiter, Claerwen Snell, Benjamin Stahl, Jerzy P Szaflarski, Shirley A Thomas, Mieke van de Sandt-Koenderman, Ineke van der Meulen, Evy Visch-Brink, Linda Worrall, Heather Harris Wright and in International Journal of Stroke

## Appendix 1

The RELEASE collaboration Marian C Brady^1^, Myzoon Ali^1^, Kathryn VandenBerg^1^, Linda J Williams^2^, Louise R Williams^1^, Masahiro Abo^3^, Frank Becker^4^, Audrey Bowen^5^, Caitlin Brandenburg^6^, Caterina Breitenstein^7^, Stefanie Bruehl^5,8^, David A Copland^6^, Tamara B Cranfill^9^, Marie di Pietro-Bachmann^10^, Pamela Enderby^11^, Joanne Fillingham^12^, Federica Lucia Galli^13^, Marialuisa Gandolfi^14^, Bertrand Glize^15^, Erin Godecke^16^, Neil Hawkins^17^, Katerina Hilari^18^, Jacqueline Hinckley^19^, Simon Horton^20^, David Howard^21^, Petra Jaecks^22^, Elizabeth Jefferies^23^, Luis MT Jesus^24^, Maria Kambanaros^25^, Eun Kyoung Kang^26^, Eman M Khedr^27^, Anthony Pak-Hin Kong^28^, Tarja Kukkonen^29^, Marina Laganaro^10^, Matthew A Lambon Ralph^30^, Ann Charlotte Laska^31^, Béatrice Leemann^32^, Alexander P Leff^33^, Roxele R Lima^34^, Antje Lorenz^35^, Brian MacWhinney^36^, Rebecca Shisler Marshall^37^, Flavia Mattioli^38^, İlknur Maviş^39^, Marcus Meinzer^40^, Reza Nilipour^41^, Enrique Noé^42^, Nam-Jong Paik^43^, Rebecca Palmer^11^, Ilias Papathanasiou^44^, Brigida Patricio^45^, Isabel Pavão Martins^46^, Cathy Price^33^, Tatjana Prizl Jakovac^47^, Elizabeth Rochon^48^, Miranda L Rose^49^, Charlotte Rosso^50^, Ilona Rubi-Fessen^51^, Marina B Ruiter^52^, Claerwen Snell^53^, Benjamin Stahl^54^, Jerzy P Szaflarski^55^, Shirley A Thomas^56^, Mieke van de Sandt-Koenderman^57^, Ineke van der Meulen^58^, Evy Visch-Brink^58^, Linda Worrall^6^, Heather Harris Wright^59^.

1. Glasgow Caledonian University, UK
2. University of Edinburgh, UK
3. The Jikei University School of Medicine, Japan
4. University of Oslo, Sunnaas Rehabilitation Hospital, Norway
5. MAHSC, University of Manchester, UK
6. The University of Queensland, Australia
7. University of Muenster, Germany
8. St Mauritius Rehabilitation Centre, and RWTH Aachen University, Germany
9. Eastern Kentucky University, USA
10. University Hospital and University of Geneva, Switzerland
11. University of Sheffield, UK
12. NHS Improvement, London, UK
13. Marche Polytechnic University, Italy
14. University of Verona, Italy
15. University of Bordeaux, France
16. Edith Cowan University, Australia
17. University of Glasgow, UK
18. City, University of London, UK
19. Nova Southeastern University, USA
20. University of East Anglia, UK
21. Newcastle University, UK
22. Bielefeld University, Germany
23. University of York, UK
24. University of Aveiro, Portugal
25. Cyprus University of Technology, Cyprus
26. Kangwon National University Hospital, Republic of Korea
27. Assiut University Hospital, Egypt
28. University of Hong Kong, Hong Kong
29. Tampere University Hospital, Finland
30. University of Cambridge, UK
31. Karolinska Institutet, Sweden
32. Hôpitaux Universitaires de Genève, Switzerland
33. University College London, UK
34. Educational Association Bom Jesus—IELUSC, Brazil
35. Humboldt University Berlin, Germany
36. Carnegie Mellon University, USA
37. University of Georgia, USA
38. Azienda Socio Sanitaria Territoriale, Italy
39. Anadolu University, Turkey
40. University Medicine Greifswald, Germany
41. University of Social Welfare and Rehabilitation Sciences, Iran
42. NEURORHB-Hospitales Vithas, Spain
43. Seoul National University College of Medicine, Republic of Korea
44. University of Patras, Patras, Greece
45. School of Polytechnic Institute of Porto, Portugal
46. Universidade de Lisboa, Portugal
47. University of Zagreb, Croatia
48. University of Toronto, Canada
49. La Trobe University, Australia.
50. Sorbonne Université, and Hôpital Salpetriere, France
51. RehaNova Rehabilitation Hospital and University of Cologne, Germany
52. Sint Maartenskliniek and Radboud University, Netherlands
53. Warrington and Halton NHS Foundation Trust, UK
54. Charité Universitätsmedizin Berlin, Germany
55. University of Alabama at Birmingham, USA
56. University of Nottingham, UK
57. University Medical Center Rotterdam, Netherlands
58. Erasmus University Medical Center, Netherlands
59. North Carolina University, USA.
