# Supplementary material for: Precision rehabilitation for aphasia by patient age, sex, aphasia severity, and time since stroke? A prespecified, systematic review-based, individual participant data, network, subgroup meta-analysis
Source: Int J Stroke. 2022 May 18;17(10):1067–77. doi: 10.1177/17474930221097477 (PMC9679795; doi:10.1177/17474930221097477)
Supplement: sj-docx-1-wso-10.1177_17474930221097477 – Supplemental material for Precision rehabilitation for aphasia by patient age, sex, aphasia severity, and time since stroke? A prespecified, systematic review-based, individual participant data, network, subgroup meta-analysis [file sj-docx-1-wso-10.1177_17474930221097477.docx]

## **Precision rehabilitation for aphasia by patient age, sex, aphasia severity, and time since stroke? A prespecified, systematic review based, individual participant data, network, subgroup meta-analysis**

# SUPPLEMENTAL MATERIAL

| **Table of Contents** | | **Page** |
| --- | --- | --- |
| Supplemental Material A | IPD subgroup network meta-analysis; intervention regimen categories, subgroups and outcomes | 3 |
| Supplemental Material B | Additional Methodological Detail: Risk of bias | 4 |
| Supplemental Material C | PRISMA Flow diagram; data searching and identification | 5 |
| Supplemental Material D | References to included randomised controlled trials | 6 |
| Supplemental Material E | Characteristics of included randomised controlled trials | 8 |
| Supplemental Material F | Characteristics of included speech and language therapy by randomised controlled trial | 12 |
| Supplemental Material G | Participant demographics | 20 |
| Supplemental Material H | **Younger (≤65 years) and Older (>65 years) subgroups by SLT frequency, intensity and dosage and language outcome** | 21 |
|  | 1. Frequency and overall language ability |  |
|  | 1. Frequency and auditory comprehension |  |
|  | 1. Frequency and functional communication |  |
|  | 1. Intensity and overall language ability |  |
|  | 1. Intensity and auditory comprehension |  |
|  | 1. Intensity and functional communication |  |
|  | 1. Dosage and overall language ability |  |
|  | 1. Dosage and auditory comprehension |  |
|  | 1. Dosage and functional communication |  |
| Supplemental Material I | **Early (≤ 3 months) and Later (>3 months) after aphasia onset; subgroups by SLT frequency, intensity and dosage and language outcome** | 30 |
|  | 1. Frequency and overall language ability |  |
|  | 1. Frequency and auditory comprehension |  |
|  | 1. Frequency and functional communication |  |
|  | 1. Intensity and overall language ability |  |
|  | 1. Intensity and auditory comprehension |  |
|  | 1. Intensity and functional communication |  |
|  | 1. Dosage and overall language ability |  |
|  | 1. Dosage and auditory comprehension |  |
|  | 1. Dosage and functional communication |  |

| Supplemental Material J | **Aphasia Severity; Below the median (Moderate-Severe) v above the median (Mild-Moderate): subgroups by SLT frequency, intensity and dosage and language outcome** | 39 |
| --- | --- | --- |
|  | 1. Frequency and overall language ability |  |
|  | 1. Frequency and auditory comprehension |  |
|  | 1. Frequency and functional communication |  |
|  | 1. Intensity and overall language ability |  |
|  | 1. Intensity and auditory comprehension |  |
|  | 1. Intensity and functional communication |  |
|  | 1. Dosage and overall language ability |  |
|  | 1. Dosage and auditory comprehension |  |
|  | 1. Dosage and functional communication |  |
| Supplemental Material K | **Male and Female subgroups by SLT frequency, intensity and dosage and language outcome** | 48 |
|  | 1. Frequency and overall language ability |  |
|  | 1. Frequency and auditory comprehension |  |
|  | 1. Frequency and functional communication |  |
|  | 1. Intensity and overall language ability |  |
|  | 1. Intensity and auditory comprehension |  |
|  | 1. Intensity and functional communication |  |
|  | 1. Dosage and overall language ability |  |
|  | 1. Dosage and auditory comprehension |  |
|  |  |  |
| Supplemental Material L | **Subgroups by language outcome and median SLT frequency, intensity, and dosage** | 57 |
| Supplemental Material M | **Base models by age, time since aphasia onset, aphasia severity and sex.** | 58 |
| Supplemental Material N | Additional Methodological Details  1. Risk of bias 2. Heterogeneity | 61 |
| Supplemental Material O | **Contributions, Declarations, role of funder and data availability** | 63 |
| Supplemental Material P | **Acknowledgements** | 65 |

### Supplementary Material A. IPD subgroup network meta-analysis; intervention regimen categories, subgroups and outcomes

|  | Frequency  *SLT days weekly* | Duration  *total SLT weeks* | Intensity  *SLT hours weekly* | Dosage  *total SLT hours* |
| --- | --- | --- | --- | --- |
|  | Up to 2 | Up to 2* | Up to 2 | Up to 5 |
|  | 3 | 3* | >2 to 3 | >5 to 14 |
| **Network Categories** | 4 | 4-10 | >3 to 4 | >14 to 20 |
|  | 5 | >10-20 | >4 to 9 | >20 to <50 |
|  | 6+ | 20+ | 9+ | 50-100 |
|  | **Age (years)** | **TSO (months)** | **Baseline severity** | **Sex** |
| **Subgroups** | ≤65 | ≤3 | Mild-moderate | Female |
|  | > 65 | >3 | Moderate-severe | Male |
|  | **Severity** | **Overall Language *WAB-AQ***  **(n)** | **Auditory Comprehension *TT-AAT***  **(n)** | **Functional Communication *AAT-SSC***  **(n)** |
| **Outcome (median)** | Mild-moderate | ≥ 64.9 (163) | ≥ 35 (145) | >2 (n=253) |
|  | Moderate-severe | < 64.9 (319) | < 35 (n = 395) | <2 (n=280) |

**Key:** * functional communication categories grouped as “up to 4 weeks”. TSO time since aphasia onset; Mild-moderate-severe n = IPD available for base model. SLT speech and language therapy; WAB-AQ Western Aphasia Battery Aphasia Quotient; TT-AAT Token Test from the Aachen Aphasia Test; AAT-SSC Aachen Aphasia Test Spontaneous Speech Communication.

### Supplementary Material B

#### Additional Methodological Detail: Risk of bias

We examined RCT-based and meta-biases, and impact on our findings, including our choice of measures informing language outcomes, a random rather than the fixed-effect model (25), and the inclusion of historical datasets (pre-2000)(22). Included RCTs and IPD were rigorously checked and verified, ensuring data were valid, reliable, consistent, and as complete as possible. The clinical, methodological, and statistical heterogeneity of included trials was reviewed, and methodological differences were recorded as a risk of bias (19). Selection, performance, detection, and attrition bias were rated as low, unclear, or high risk for each RCT. Our data synthesis procedures accommodated between-study outcome differences.

Standard data-synthesis heterogeneity assessments (e.g., I^2^) were unsuitable in the context of analysis of unique participant, intervention, and outcome IPD. Instead, variance was considered throughout, comparing variability due to study differences to data variability overall. We reported where it exceeded 25% and checked datasets for undue influence or unbalanced groups. Where it exceeded 50%, we report it for completeness, but the finding was considered unreliable and excluded from our data interpretation.

Dual observer-rated functional communication outcome IPD (Therapy Outcome Measures (TOM, (26) activity and participation subtests) were available. Previously, sensitivity analysis on these data found no indication that the choice of subtest included in the meta-analysis impacted our findings (20). Our subgroup analysis included the TOMs activity data only.

### Supplementary Material C


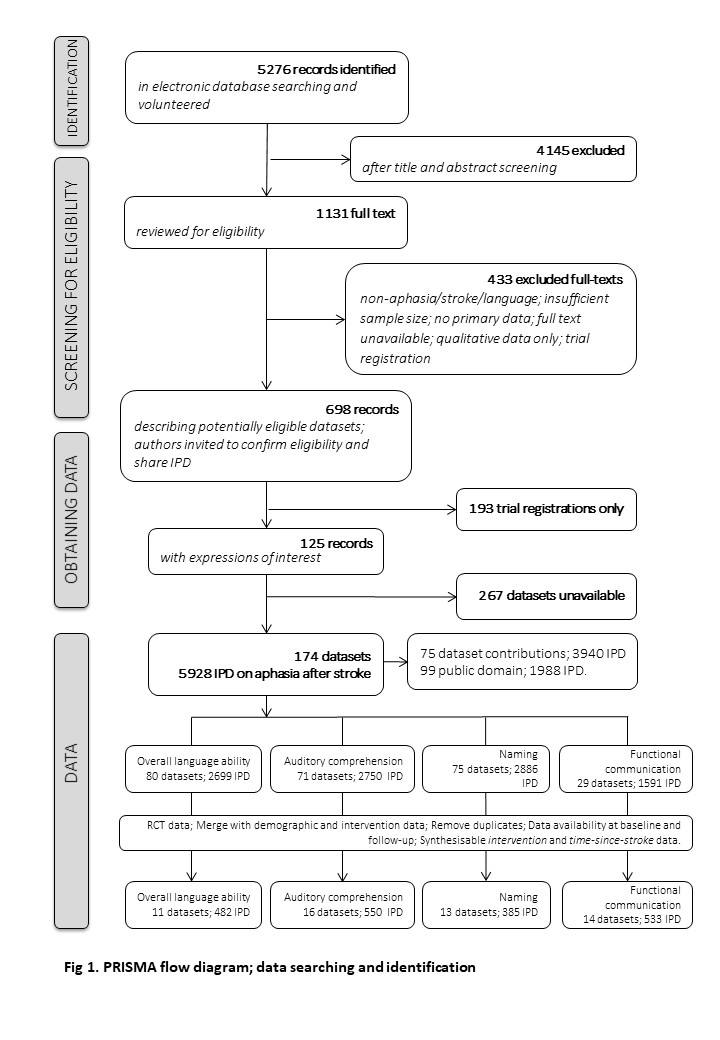


### Supplementary Material D

#### References to included randomised controlled trials

1. Breitenstein C, Grewe T, Floel A, Ziegler W, Springer L, Martus P. Intensive speech and language therapy in patients chronic aphasia after stroke: a randomised, open label, blinded-enpdpoint, controlled trial in a health care setting. The Lancet 2017; 389: 1528-1538.
2. Ciccone N, West D, Cream A, Cartwright J, Rai T, Granger A et al. Constraint-induced aphasia therapy (CIAT): a randomised controlled trial in very early stroke rehabilitation. Aphasiology 2015; 30: 566-584.
3. de Jong-Hagelstein M, van de Sandt-Koenderman WME, Prins ND, Dippel DWJ, Koudstaal PJ, Visch-Brink EG. Efficacy of early cognitive-linguistic treatment and communicative treatment in aphasia after stroke: a randomised controlled trial (RATS-2). Journal of Neurology, Neurosurgery & Psychiatry 2011; 82: 399-404.
4. Doesborgh SJC, van de Sandt-Koenderman MWME, Dippel DWJ, van Harskamp F, Koudstaal PJ, Visch-Brink EG. Cues on request: the efficacy of multicue, a computer program for wordfinding therapy. Aphasiology 2004; 18: 213-222.
5. Doesborgh SJC, van de Sandt-Koenderman MWE, Dippel DWJ, van Harskamp F, Koudstaal PJ, Visch-Brink EG. Effects of Semantic Treatment on Verbal Communication and Linguistic Processing in Aphasia After Stroke: A Randomized Controlled Trial. Stroke 2004; 35: 141-146.
6. Efstratiadou EA, Papathanasiou I, Holland R, Varlokosta S, Hilari K. Efficacy of elaborated semantic features analysis in aphasia: a quasi-randomised controlled trial. Aphasiology 2019; https://doi.org/10.1080/02687038.2019.1571558.
7. Godecke E, Hird K, Lalor EE, Rai T, Phillips MR. Very early poststroke aphasia therapy: a pilot randomized controlled efficacy trial. International Journal of Stroke 2012; 7(8): 635-644.
8. Khedr EM, Abo El-Fetoh N, Ali AM, El-Hammady DH, Khalifa H, Atta H et al. Dual-hemisphere repetitive transcranial magnetic stimulation for rehabilitation of poststroke aphasia: a randomized, double-blind clinical trial. Neurorehabilitation and Neural Repair 2014; 28: 740-750.
9. Kukkonen T and Korpijaakko-Huuhka AM. How much is enough and when is the right time? What do we know about the good practice and timing of aphasia rehabilitation? Edinburgh, UK: British Aphasiology Society; 2007.
10. Laska AC, Kahan T, Hellblom A, Murray V, von Arbin M. A randomized controlled trial on very early speech and language therapy in acute stroke patients with aphasia. Cerebrovascular Diseases Extra. 2011; 1: 66-74.
11. Lincoln NB. *An investigation of the effectiveness of language retraining methods with aphasic stroke patients*. PhD Thesis. London: University of London; 1980.
12. Martins IP, Leal G, Fonseca I, Farrajota L, Aguiar M, Fonseca J et al. A randomized, rater-blinded, parallel trial of intensive speech therapy in sub-acute post-stroke aphasia: the SP-I-R-IT study. International Journal of Language and Communication Disorders 2013; 48: [https://doi.org/10.1111/1460-6984.12018].
13. Mattioli F, Ambrosi C, Mascaro L, Scarpazza C, Pasquali P, Frugoni M et al. Early aphasia rehabilitation is associated with functional reactivation of the left inferior frontal gyrus a pilot study. Stroke 2014; 45: 545-552.
14. Meikle M, Wechsler E, Tupper A, Benenson M, Butler J, Mulhall D et al. Comparative trial of volunteer and professional treatments of dysphasia after stroke. British Medical Journal 1979; 2(6182), 87-89.
15. Meinzer M, Streiftau S, Rockstroh B. Intensive language training in the rehabilitation of chronic aphasia: efficient training by laypersons. Journal of the International Neuropsychological Society 2007; 13: 1-8.
16. Palmer R, Enderby P, Cooper C, Latimer N, Julious S, Paterson G et al. Computer Therapy Compared With Usual Care for People With Long-Standing Aphasia Poststroke: A Pilot Randomized Controlled Trial. Stroke 2012; 43: 1904-1911.
17. Rodriguez AD, Worrall L, Brown K, Grohn B, McKinnon E, Pearson C et al. Aphasia LIFT: exploratory investigation of an intensive comprehensive aphasia programme. Aphasiology 2013; 27: 1339-1361.
18. Rubi-Fessen I, Hartmann A, Huber W, Fimm B, Rommel T, Thiel A et al. Add-on effects of repetitive transcranial magnetic stimulation on subacute aphasia therapy: enhanced improvement of functional communication and basic linguistic skills. A randomized controlled study. Archives of Physical Medicine and Rehabilitation 2015; 96: 1935-1944.
19. Szaflarski JP, Ball AL, Vannest J, Dietz AR, Allendorfer JB, Martin AN et al. Constraint-induced aphasia therapy for treatment of chronic post-stroke aphasia: a randomized, blinded, controlled pilot trial. Medical Science Monitor, 2015;21:1643-3750.
20. Smania N, Aglioti SM, Girardi F, Tinazzi M, Fiaschi A, Casentino A et al. Rehabilitation of limb apraxia improves daily life activities in patients with stroke. Neurology 2006; 67: 2050-2052.
21. Smania N, Girardi F, Domenicali C, Lora E, Aglioti S. The rehabilitation of limb apraxia: a study in left-brain-damaged patients. Archives of Physical Medicine and Rehabilitation 2000; 81: 379-388.
22. Van Der Meulen I, van de Sandt-Koenderman MWME, Heijenbrok MH, Visch-Brink E, Ribbers GM. Melodic Intonation Therapy in chronic aphasia: evidence from a pilot randomized controlled trial. Frontiers in Human Neuroscience 2016; 10(533): https://doi.org/10.3389/fnhum.2016.00533.
23. Woodhead ZVJ, Crinion J, Teki S, Penny W, Price CJ, Leff AP. Auditory training changes temporal lobe connectivity in 'Wernicke's aphasia': a randomised trial. Journal of Neurology, Neurosurgery & Psychiatry 2017; 88: 586-594.
24. You DS, Kim D-Y, Chun MH, Jung SE, Park SJ. Cathodal transcranial direct current stimulation of the right Wernicke's area improves comprehension in subacute stroke patients. Brain and Language 2011; 119: 1-5.

### Supplementary Material E

#### Characteristics of included randomised controlled trials

| **Primary Publication reference; Country; Funder** | **Participants’ inclusion and exclusion criteria** | **IPD; data time-points; electronic or public domain in RELEASE** |
| --- | --- | --- |
| Ciccone (2015)  Australia  Funder Unreported | Inclusion: stroke (less than 5 days); aphasia (score below ceiling of WAB); teaching hospital admission; conscious and medically stable; can maintain alert state for at least 30 minutes  Exclusion: previous history of aphasia, mental illness or dementia; non-English speaking background; history of sub-arachnoid and / or subdural haemorrhage or neurosurgical intervention; uncorrected hearing or vision impairment | 20 IPD  Baseline; 3 months; 6 months  Electronic |
| de Jon-Hagelstein (2011)  The Netherlands  Stichting Nuts Ohra (T-07-71) | Inclusion: adult; stroke (less than 3 weeks); aphasia (verbal communication, semantic or phonological disorder, tests and cut-offs defined); life expectancy more than 6 months  Exclusion: over 85 years; severe dysarthria; premorbid dementia or aphasia; developmental dyslexia; visual perceptual disorder; recent psychiatric disorder | 85 IPD (75 complete)  Baseline; 3 months; 6 months  Electronic |
| Doesborgh (2004a)  The Netherlands  Netherlands Organisation for Scientific Research | Inclusion: adult (age 20 to 86); stroke (at least 11 months); aphasia (moderate to severe naming deficit BNT); completed intensive impairment-oriented (semantic or phonological) therapy; native speaker (Dutch)  Exclusion: global or minimal aphasia; dysarthria; non-native Dutch speaker; illiteracy, developmental dyslexia, severe acquired dyslexia; visual perceptual deficit | 18 IPD  Baseline; 2 months  Electronic |
| Doesborgh (2004b)  The Netherlands  Netherlands Organization for Health Research and Development, Chronic Diseases (940-33- 008) | Inclusion: adult; stroke; aphasia (moderate or severe; both semantic and phonological deficit); one of 35 clinical centres; speech and language therapist considered a candidate for intensive treatment (taking into account practical, psychological, physical, cognitive factors);  Exclusion: within 3 months of onset; dysarthria; global aphasia; recovered aphasia; non-native speaker; illiteracy; developmental dyslexia; severe acquired dyslexia; visual perceptual deficit | 58 IPD  Baseline; 11 months  Electronic |
| Mattioli (2014)  Italy  Funder Unreported | Inclusion: adult; stroke (first, acute); aphasia with mildly impaired comprehension; native speaker (Italian); suitable for MRI; right-handed; no other neurological or psychiatric disease; no hearing deficit  Exclusion: over 80 years; stroke not in middle cerebral artery; aphasia with severely impaired comprehension; not native Italian speaker; unsuitable for MRI (pacemaker; claustrophobia; severe obesity); dementia; psychiatric disorders; deafness | 12 IPD  Baseline; 16 days; 190 days  Electronic |
| Meikle (1979)  UK  Chest, Heart, and Stroke Association | Inclusion: stroke (at least 3 weeks); aphasia (less than 4^th^ percentile on PICA); previously proficient in English; well enough to attend  Exclusion: dementia; lives too far from hospital | 31 IPD  Baseline; 4, 15, 24, 35, 42, 66, 84 weeks  Public domain |
| Laska (2011)  Sweden  Stockholm County Council Foundation (Expo-95); AFA Insurances; Marianne and Marcus Wallenberg Foundation; Karolinska Institute | Inclusion: stroke (first); aphasia (NGA 0 to 59); able to start SLT within 2 days of onset  Exclusion: rapid regression; dementia; drug abuse; severe illness; unable to participate in treatment (as judged by investigator) | 125 IPD (plus 2 without group allocation)  Baseline; 3 weeks (16 days); 6 months  Electronic |
| Rodriguez (2013)  Australia  National Health and Medical Rehabilitation Council Centre for Clinical Research Excellence in Aphasia Rehabilitation (Grant 569935); DC was funded by an Australia Research Council Future Fellowship and NHMRC Career Development Fellowship | Inclusion: stroke (at least 6 months); aphasia; no other neurological disorders; sufficient vision and hearing to take part  Exclusion: concomitant neurological illness | 11 IPD  Baseline; 2 weeks; 4 weeks; 9 weeks;11 weeks  Electronic |
| Woodhead (2017)  UK  Wellcome Trust and the James S McDonnell Foundation, personal fellowships from the Wellcome Trust (ME033459MES and 106084/Z/14/Z). | Inclusion: adult; stroke (3 or more months); aphasia (Wernicke’s); competent to consent  Exclusion: under 18; significant medical or psychiatric co-morbidity; unable to comply with treatment regime or scanning; significant multifocal cerebral disease; contraindications to cholinesterase inhibitors (sick sinus syndrome; pregnancy; lactation); contraindications to fMRI and MEG (pacemaker; noncompatible metallic implant); severe hearing impairment; unable to provide informed consent | 20 IPD  Baseline; 5 weeks; 10 weeks  Electronic |
| Lincoln (1980a)  UK  Funder unreported | Inclusion: adult; stroke; no other brain damage; aphasia; referred for SLT by medical staff; able to attend daily (4 days per week) for 8 weeks as in- or out-patient  Exclusion: severely or mildly aphasic | 24 IPD  Baseline; week 4; week 8  Public domain |
| Lincoln (1980b)  UK  Funder unreported | Inclusion: adult; stroke; no other brain damage; severe aphasia; referred for SLT by medical staff; able to attend daily (4 days per week) for 8 weeks as in- or out-patient  Exclusion: unreported | 24 IPD  Baseline; week 4; week 8  Public domain |
| Szaflarski (2015)  USA  NINDS R01 NS 048281 and by NIH/NCRR UL1-RR026314 (REDCap Database) | Inclusion: stroke (single); aphasia (chronic)  Exclusion: more than one stroke; history degenerative or metabolic disorder or supervening illness; history depression or other mental illness; pregnant | 24 IPD  Baseline; 2 weeks; 12 weeks  Electronic |
| Palmer (2012)  UK  NIHR Research for Patient Benefit (RfPB) Programme (Grant no. PB-PG-1207-14097) | Inclusion: stroke; aphasia (predominant word-finding difficulties; able to repeat spoken words); ceased impairment-focused SLT; motor deficits if co-existing; upper limb impairment if computer access addressed by assistive devices  Exclusion: severe visual or cognitive difficulties | 34 IPD  Baseline; 5 months; 8 months  Electronic |
| Smania (2006) and (2000)  Italy  Ministero Italiano Universita’ Ricerca and Finanziamento Italiano Ricerca di Base (FIRB) both awarded to Salvatore M. Aglioti; M.U.R.S.T. and the Consiglio Nazionale delle Ricerche, Italy | Inclusion: stroke; aphasia; limb apraxia (ideational or ideomotor) for at least 2 months  Exclusion: history of stroke or other neurological disorders; over 80 years; uncooperativeness; orthopedic or other disabling disorders | 32 IPD  Baseline; 10 weeks  Electronic |
| Breitenstein (2017)  Germany  German Federal Ministry of Education and Research; German Society for Aphasia Research and Treatment | Inclusion: adult; stroke; aphasia for at least 6 months; native speaker (German); at least basic level of communication and language comprehension  Exclusion: severe untreated medical conditions; severe uncorrected vision or hearing impairments; aphasia from traumatic brain injury or neurodegenerative disease; participation in any intensive stroke intervention in previous 4 weeks | 142 (minus14)  Screening; baseline; 3 weeks; 6 weeks (subgroup only); 6 months  Electronic |
| Godecke (2012)  Australia  Unfunded | Inclusion: stroke (acute); aphasia (less than 5 days; score of 13 or less on FAST); admitted to teaching hospital; conscious, medically stable, able to maintain alertness for at least 30 minutes  Exclusion: previous history subarachnoid/subdural haemorrhage, neurosurgical intervention, aphasia, mental illness, dementia; non-English speaking; uncorrected hearing or vision impairment; already 3 participants in daily therapy group | 59 IPD  Baseline; 4 weeks (or acute hospital discharge if sooner); 6 months  Electronic |
| Kukkonen (unpublished)  Finland  Unfunded | Inclusion: older adult (50-64; 65-80); stroke (first); aphasia; right-handed; living in Tampere with someone; no dementia; normal hearing and vision  Exclusion: age under 50; two or more, right hemisphere, or haemorrhagic stroke; dementia or other neurological disease; left-handed; living alone; living outside Tampere; problems with hearing or vision | 36 IPD  Baseline; 4 weeks; 10 weeks; 14 weeks; 20 weeks; 32 weeks; 56 weeks  Unpublished |
| Martins (2013)  Portugal  Funder unreported | Inclusion: adult (40-80); stroke (single); aphasia (LAAB mild/moderate and severe); native speaker (Portuguese); willing to participate  Exclusion: more than 3 months since stroke or further stroke; very severe or very mild aphasia; illiteracy; unable to attend on daily basis; evidence of dementia or other severe medical or psychiatric disorder; miss more than 5 consecutive hours of intervention | 30 IPD (14 complete)  Baseline; 10 weeks; 50 weeks; 62 weeks  Electronic |
| Meinzer (2007)  Germany  Deutsche Forschungsgemeinschaft (Grant RO 805011-4), the Kuratorium Zentrales Nervensystem (Grant 2001013) | Inclusion: stroke (single); aphasia (at least 6 months; global aphasia if residual expressive language); 1 or more participating relative  Exclusion: well-recovered people with minimal aphasia symptoms | 20 IPD  Baseline; 10 days  Electronic |
| Khedr (2014)  Egypt  Funder unreported | Inclusion: stroke (single); aphasia (non-fluent); subacute hemiplegia  Exclusion: head injury or neurological disease other than stroke; unstable cardiac dysrhythmia; fever; infection; hyperglycemia; prior administration of tranquiliser; safety contraindications for rTMS | 29 IPD  Baseline; 2 weeks; 6 weeks; 10 weeks  Electronic |
| van der Meulen (2016)  The Netherlands  Stichting Rotterdams Kinderrevalidatie Fonds Adriaanstichting (Grant 2007/0168 JKF/07.08.31 KFA). | Inclusion: adult; stroke (more than 1 year); aphasia (candidate for MIT: non-fluent; poor language repetition; poorly articulated speech; moderate to good auditory comprehension)  Exclusion: prior stroke resulting in aphasia; bilateral lesion; intensive MIT prior to start of study; severe hearing deficit; relevant psychiatric history | 17 IPD  Baseline; 42 days; 82 days  Electronic |
| Rubi-Fessen (2015)  Germany  Walter and Marga Boll Foundation and the Wolf-Dieter Heiss-Foundation | Inclusion: 55 to 85 years; stroke (first; up to 16 weeks); aphasia; first language (German); right-handed  Exclusion: previous stroke, neurodegenerative or psychiatric disease; epilepsy; auditory or visual deficits that might impair testing | 30 IPD  Baseline; 2 weeks  Electronic |
| Efstratiadou (2019)  Greece  European Social Fund, EFSA, National Strategic Reference Framework —Research Funding Program: THALES UOA. | Inclusion: adult; stroke (at least 4 months); aphasia; native speaker (Greek); medically stable; no other neurological or psychiatric history; no considerable cognitive impairment  Exclusion: in receipt of other SLT during the project; not living independently at home prior to the stroke  Not in RELEASE: 20 received alternative SLT | 38 IPD  Baseline; 19 weeks; 32 weeks  Electronic |
| You (2011)  Korea  Funder unreported | Inclusion: stroke; not taking pharmacological drugs  Exclusion: history of previous stroke, seizure, multiple stroke lesions; metal implants in brain; taking certain medication; uncooperative with SLT | 21 IPD  Baseline; 2 weeks  Electronic |

### Supplementary Material F

#### Characteristics of included speech and language therapy interventions by randomised controlled trial

| **Primary Publication reference** | **Location** | **Group** | **Therapy Impairment Target:** | **Theoretical Approach:** | **Provided by:** | **Delivery:** | **Regimen:** | | **Tailoring:** |  |
| --- | --- | --- | --- | --- | --- | --- | --- | --- | --- | --- |
| **Mattioli (2014)** | Hospital, then outpatient | **Group 1:** n=6 | Mixed SLT and Word Finding SLT | Unreported | Speech and language therapist | Face-to-face; 1-to-1 | Frequency: 5 days per week. Duration: 2 months. Intensity: 5 hours. Dosage: 10 hours. | | Unreported |  |
|  |  | **Group 2:** n=6 No SLT | | | | | | | |  |
| **Meikle (1979)** | Home and groups at rehabilitation centre | **Group 1:** n=16  “Conventional SLT” | Unreported | Unreported | Speech and language therapist | face-to-face; 1-to-1 and group; | Frequency: 3-5 days per week. Duration: IPD. Intensity: between 2 hours 15 minutes and 3 hours 45 minutes. Dosage: IPD | | Unreported |  |
|  |  | **Group 2:** n=15  “Conventional SLT” | Mixed SLT | Unreported | recruited volunteers. | face-to-face; 1-to-1 and group; | Frequency: 4 home visits per week and a separate group session at rehabilitation centre. Duration: IPD. Intensity: between 2 hours 15 minutes and 3 hours 45 minutes. Dosage: IPD | | Difficulty |  |
| **Laska (2011)** | Stroke unit, or discharged to (home, rehabilitation clinic, geriatric clinic, nursing home). | **Group 1:** n=62 | Mixed SLT | Unreported | Speech and language therapist | Face-to-face; 1-to-1 | Frequency: 3 sessions each day 5 days per week. Duration: 3 weeks. Intensity: 3 hours 45 minutes. Dosage: 11 hours 15 minutes. | | Functional relevance |  |
|  |  | **Group 2:** n=61 Intervention type(s): No SLT | | | | | | | |  |
| **Rodriguez (2013)** | Aphasia clinic and other rehabilitation centres. | **Group 1:** n=4 | Word Finding SLT and Mixed SLT | Functional or Pragmatic SLT; Semantic and Phonological SLT | speech and language therapists and students. | face-to-face; 1-to-1 and group; | Frequency: 5 days per week. Duration: 2 weeks. Intensity: 20 hours. Dosage: 40 hours. Home practice reported. | | Functional relevance and difficulty |  |
|  |  | **Group 2:** n=7 | Word Finding SLT and Mixed SLT | Functional or Pragmatic SLT; Semantic and Phonological SLT | speech and language therapists and students. | face-to-face and computer-based treatment; 2-to-1 and group; | Frequency: 5 days each week. Duration: 4 weeks. Intensity: 25 hours. Dosage: 100 hours. Home practice reported. | | Functional relevance and difficulty |  |
| **Woodhead (2017)** | Home | **Group 1:** n=14  Intervention type(s): SLT intervention | Auditory Comprehension SLT | Phonological SLT plus Co-intervention (Donepezil) | experimental psychologist. | computer-based; self-managed; | Frequency: 7 days a week. Duration: 25 weeks in study, but intervention is over two 5-week blocks. Intensity: 7.3 hours (according to diaries) on average. Dosage: 73 hours (according to diaries). Home practice reported. | | Difficulty |  |
|  |  | **Group 2:** n=13  Intervention type(s): SLT intervention  Delivery: Location: Regimen: 10 hours of training per week over each 5 week training block. | Auditory Comprehension SLT | Phonological SLT plus Co-intervention (placebo) | experimental psychologist | computer-based; self-managed; | Frequency: 7 days a week. Duration: 25 weeks in study, but intervention is over two 5-week blocks. Intensity: 7.3 hours (according to diaries) on average. Dosage: 73 hours (according to diaries). Home practice reported. | | Difficulty |  |
| **Lincoln (1980a)** | Hospital and home | **Group 1:** 6 | Mixed SLT | Unreported | Speech and language therapist | Face-to-face; 1-to-1 | Frequency: 4 days per week. Duration: 3.5 weeks. Intensity: 2 hours. Dosage: 7 hours. | | Unreported |  |
|  |  | **Group 2:** 7  No SLT (operant training) then Conventional SLT | Mixed SLT | Unreported | Speech and language therapist | Face-to-face; 1-to-1 | hospital and ho Frequency: 4 days per week. Duration: 3.5 weeks. Intensity: 2 hours. Dosage: 7 hours. | | Unreported |  |
|  |  | **Group 3**: n=5  Intervention type(s): Social Support then Conventional SLT | Mixed SLT | Unreported | Speech and language therapist | Face-to-face; 1-to-1 | Frequency: 4 days per week. Duration: 3.5 weeks. Intensity: 2 hours. Dosage: 7 hours. | | Unreported |  |
|  |  | **Group 4:** n=6 | Mixed SLT | Unreported | Speech and language therapist | Face-to-face; 1-to-1 | Frequency: 4 days per week. Duration: 3.5 weeks. Intensity: 2 hours. Dosage: 7 hours. | | Unreported |  |
| **Lincoln (1980b)** | Hospital | **Group 1:** n=12 | Operant training with SLT then Social Support with SLT | Mixed SLT | Speech and language therapist and psychologist | Face-to-face; 1-to-1 | Frequency: IPD between 1.25 and 3.5 days per week. Duration: 8 weeks. Intensity: 2 hours per week. Dosage: IPD. | | Difficulty |  |
|  |  | **Group 2:** n=12 | SLT with Social Support, then operant training with SLT  Mixed SLT | Unreported | Speech and language therapist and psychologist | Face-to-face; 1-to-1 | Frequency: IPD between 1.25 and 3.5 days per week. Duration: 8 weeks. Intensity: 2 hours per week. Dosage: IPD | | Difficulty |  |
| **Szaflarski (2015)** | Hospital | **Group 1:** n=14 | Word-finding SLT; Spoken Language SLT | Constraint Induced Aphasia Therapy | Speech and language therapist | face-to-face; groups of 3 to 4; | Frequency: 5 times per week; Duration: 2 weeks; Intensity: 20 hours. Dosage: 40 hours. | | Difficulty |  |
|  |  | **Group 2:** n=10 | Intervention type(s): No SLT | | | | | | |  |
| **Palmer (2012)** |  | **Group 1:** n=16 | Word-finding SLT and Mixed SLT | Unreported | Self-managed, computer software, supported by speech and language therapist, volunteer. | Home visit plus computer or phone call plus computer; 1-to-1; | Frequency: IPD. Duration**:** 5 months. Intensity: IPD. Dosage: IPD | | Functional relevance |  |
|  |  | **Group 2:** n=17 | Intervention type(s): No SLT | | | | | |  |  |
| **Smania (2006) and (2000)** | Therapy clinic | **Group 1:** n=17 | Intervention type(s): No SLT (limb apraxia therapy only) | | | | | | |  |
|  |  | **Group 2:** n= 15 | unreported | unreported | Speech and language therapist | unreported; | Frequency: 3 days per week. Duration:10 weeks. Intensity: 2.5 hours. Dosage: 25 hours. | | Unreported |  |
| **Breitenstein (2017)** | Inpatient and outpatient rehabilitation | **Group 1:**  N=78 | Mixed SLT | Functional or Pragmatic SLT | Speech and language therapist | face-to-face; 1-to-1 and group | Frequency: IPD. Duration: IPD.  Intensity: IPD.  Dosage: IPD. Home practice reported. | | Difficulty |  |
|  | Outpatient | **Group 2:** n=78 | Unreported (usual care) | Unreported | Speech and language therapist | face-to-face; 1-to-1 and group | Frequency: IPD.  Duration: 3 weeks  Intensity: IPD. Dosage: IPD. | | Unreported |  |
| **Godecke (2012)** | Hospital or rehabilitation | **Group 1:** n=32 | Spoken language SLT | Semantic and Phonological SLT | Speech and language therapist | Face-to-face; 1-to-1 | Frequency: 5 days per week. Duration: IPD but maximum of 1 month. Intensity: IPD between 2.5 and 7.5 hours per week. Dosage: IPD up to 26.5 hours. | | Functional relevance and difficulty |  |
|  |  | **Group 2:** n=27 | Spoken Language SLT | Semantic and Phonological SLT | Speech and language therapist | Face-to-face; 1-to-1 | Frequency: 1 day per week. Duration: IPD up to 1 month. Intensity: up to 1.5 hours per week. Dosage: IPD up to 5.3 hours. | | Functional relevance and difficulty |  |
| **Ciccone (2015)*** | Hospital, rehabilitation or home | **Group 1:** n=8 | Word Finding SLT | Phonological and Semantic SLT | Speech and language therapist | Face-to-face; 1-to-1 | Frequency: IPD. Duration: 5 weeks. Intensity: IPD. Dosage: IPD. | | Functional relevance and difficulty |  |
|  |  | **Group 2:** n=12 | Word Finding SLT | Phonological and Semantic SLT; Constraint Induced Aphasia Therapy. | Speech and language therapist | face-to-face; group; | Frequency: IPD. Duration: 5 weeks. Intensity: IPD. Dosage: IPD. | | Functional relevance and difficulty |  |
| **Kukkonen (unpublished)** | SLT clinic | **Group 1:** n=9 | Mixed SLT | Language Enrichment Therapy | Speech and language therapist | Face-to-face; 1-to-1 | Frequency: 5 days per week. Duration: 6 weeks + 6 weeks. Intensity: 10 hours. Dosage: 120 hours. | | Functional relevance |  |
|  |  | **Group 2:** n=8 | Mixed SLT | Language Enrichment Therapy | Speech and language therapist | Face-to-face; 1-to-1 | Frequency: 2 days per week. Duration: 6 weeks + 6 weeks. Intensity: 2 hours. Dosage: 48 hours | | Functional relevance |  |
|  |  | **Group 3:** n=10 | Mixed SLT | Language Enrichment Therapy | Speech and language therapist | Face-to-face; 1-to-1 | Frequency: 1 day per week. Duration: 6 weeks + 6 weeks. Intensity: 1 hour. Dosage: 24 hours. | | Functional relevance |  |
|  |  | **Group 4:** n=9 | Spouses or caregiver(s) received support and information from the speech and language therapists | | | | Twice, 1 hour per meeting | |  |  |
| **Martins (2013)** | Medical and rehabilitation centres, outpatient rehabilitation unit, acute stroke unit. | **Group 1:** n=15 | Mixed SLT | Multimodal | Speech and language therapist | Face-to-face; 1-to-1 | Frequency: 5 days per week. Duration: 10 weeks. Intensity: 10 hours. Dosage: 100 hours. Home practice reported. | | Functional relevance and difficulty |  |
|  |  | **Group 2:** n=15 | Mixed SLT | Multimodal Stimulation Approach (MSA) (Duffy 2001) | Speech and language therapist | Face-to-face; 1-to-1 | Frequency: 1 day per week. Duration: 50 weeks. Intensity: 2 hours. Dosage: 100 hours. Home practice reported. | | Functional relevance and difficulty |  |
| **Meinzer (2007)** | Unreported | **Group 1:** n=10 | Word Finding SLT | Constraint Induced Aphasia Therapy | trained psychologists | Face-to-face; group | Frequency: 5 days per week. Duration: 10 days. Intensity: 15 hours. Dosage: 30 hours. Home practice reported. | | Functional relevance and difficulty |  |
|  |  | **Group 2:** n=10 | Word Finding SLT | Constraint Induced Aphasia Therapy | Volunteer relatives with training and supervision | Face-to-face; group | Frequency: 5 days per week. Duration: 10 days. Intensity: 15 hours. Dosage: 30 hours. Home practice reported. | | Functional relevance and difficulty |  |
| **Doesborgh (2004a)** | Unreported | **Group 1:** n=8 | Word Finding SLT | Unreported | Speech and language therapist | Computer, supervised by therapist; self-managed; | Frequency: 2 days per week. Duration: 2 months. Intensity: 1 to 1.5 hours weekly. Dosage: 10 to 11 hours. | | Difficulty |  |
|  |  | **Group 2:** n=10 | No SLT | | | |  |  | | |
| **Khedr (2014)** | Hospital | **Group 1:** n=10 | Mixed SLT | Unreported | Speech and language therapist | Face-to-face; 1-to-1 | Frequency: 5 days per week. Duration: 2 weeks. Intensity: 2.5 hours. Dosage: 5 hours. | | Difficulty |  |
|  |  | **Group 2:** n=19 | Mixed SLT | Unreported | Speech and language therapist | Face-to-face; 1-to-1 | Frequency: 5 days per week. Duration: 2 weeks. Intensity: 2.5 hours. Dosage: 5 hours. | | Difficulty |  |
| **de Jon-Hagelstein (2011)** | Hospital, rehabilitation clinic, home, nursing home. | **Group 1:** n=41 | Unreported | Semantic and Phonological SLT | Speech and language therapist | Face-to-face; 1-to-1 | Frequency: 3.25 times per week on average. Duration: 6 months (or less if fully recovered). Intensity: 2 to 5 hours. Dosage: 52 hours. Home practice reported. | | Difficulty |  |
|  |  | **Group 2:** n=44 | Unreported | Functional or Pragmatic SLT | Speech and language therapist | Face-to-face; 1-to-1 | Frequency: 3.25 times per week on average. Duration: 6 months (or less if fully recovered). Intensity: 2 to 5 hours. Dosage: 52 hours. Home practice reported. | | Difficulty |  |
| **Doesborgh (2004b)** | Hospital / rehabilitation clinic / home / nursing home. | **Group 1:** n=29 | Word Finding SLT | Semantic SLT | Speech and language therapist | Face-to-face and computer; 1-to-1; | Frequency: 2.25 days a week on average. Duration: 40 weeks. Intensity: 1.5 to 3 hours. Dosage: 40 to 60 hours. Home practice reported. | | Difficulty |  |
|  |  | **Group 2:** n=29 | Word Finding SLT | Phonological SLT | Speech and language therapist | Face-to-face and computer; 1-to-1; | Frequency: 2.25 days a week on average. Duration: 40 weeks. Intensity: 1.5 to 3 hours. Dosage: 40 to 60 hours. Home practice reported. | | Difficulty |  |
| **van der Meulen (2016)** | Rehabilitation / aphasia centres. | **Group 1:** n=10 | Spoken language SLT | Melodic Intonation Therapy | Speech and language therapist | Face-to-face; 1-to-1 | Frequency: 5 days a week. Duration: 12 weeks (6 MIT and 6 no therapy). Intensity: 5 hours a week. Dosage: 30 hours. Home practice reported. | | Functional relevance and difficulty |  |
|  | Rehabilitation centre / nursing home with rehabilitation facilities. | **Group 2:** n=7 | Auditory Comprehension SLT | unreported (protocol of what was and was not permitted, and manual of practice materials and references; PI helped create tailor-made tasks for a specific participant) | speech and language therapists. | Face-to-face; 1-to-1 | Frequency: 5 days a week. Duration: 6 weeks. Intensity: 5 hours a week. Dosage: 30 hours. | | Functional relevance and difficulty |  |
| **Rubi-Fessen (2015)** | Hospital | **Group 1:** n=15  SLT intervention with rTMS | Word Finding SLT | Unreported | Speech and language therapist | Face-to-face; 1-to-1 | Frequency: 5 days a week. Duration: 2 weeks. Intensity: 3.75 hours. Dosage: 7.5 hours. | | Functional relevance and difficulty |  |
|  |  | **Group 2:** n=15  SLT intervention with sham rTMS | Word Finding SLT | Unreported | Speech and language therapist | Face-to-face; 1-to-1 | Frequency: 5 days a week. Duration: 2 weeks. Intensity: 3.75 hours. Dosage: 7.5 hours. | | Functional relevance and difficulty |  |
| **Efstratiadou (2019)** | Home and hospital | **Group 1:** n=18 | Word Finding SLT | Semantic SLT | Speech and language therapist | Face-to-face; 1-to-1 | Frequency: 3 days a week. Duration: 12 weeks. Intensity: 3 hours. Dosage: 36 hours. No home practice. | | Difficulty |  |
|  |  | **Group 2:** n=8 | Word Finding SLT | Semantic SLT | Speech and language therapist | Face-to-face; 1-to-1 | Frequency: 3 days a week. Duration: 12 weeks. Intensity: 3 hours. Dosage: 36 hours. No home practice. | | Difficulty |  |
|  |  | **Group 3:** n=12 | No SLT but then as per Group 1 (n=4) or Group 2 (n=6) above | | | | | | |  |
| **You (2011)** | Hospital rehabilitation department | **Group 1:** n=7 | Mixed SLT | Functional or Pragmatic SLT and Co-intervention anodal tDCS) | Speech and language therapist | Face-to-face; 1-to-1 | Frequency: 5 days a week. Duration: 2 weeks. Intensity: 2.5 hours. Dosage: up to 5 hours. | | Unreported |  |
|  |  | **Group 2:** n=7 | Mixed SLT | Functional or Pragmatic SLT and Co-intervention (cathodal tDCS) | Speech and language therapist | Face-to-face; 1-to-1 | Frequency: 5 days a week. Duration: 2 weeks. Intensity: 2.5 hours. Dosage: up to 5 hours. | | Unreported |  |
|  |  | **Group 3:** n=7 | Mixed SLT | Functional or Pragmatic SLT and Co-intervention (sham tDCS) | Speech and language therapist | Face-to-face; 1-to-1 | Frequency: 5 days a week. Duration: 2 weeks. Intensity: 2.5 hours. Dosage: up to 5 hours. | | Unreported |  |

### Supplementary Material G. Participant demographics

|  | **IPD**  **(RCTs)** |  | **IPD** | **Median [IQR] (%)** |  | **IPD**  **(RCTs)** |  | **IPD** | **Median [IQR] (%)** |
| --- | --- | --- | --- | --- | --- | --- | --- | --- | --- |
| **Age (years)** | 941 (24) |  | 928 | 63.0 [54.1, 74.0] | **Hemisphere lesion** | 699 (18) | Bilateral  Left  Right | 6  683  10 | (0.9)  (97.7)  (1.4) |
| **Sex** | 928 (24) | Female  Male | 390  538 | (42.0)  (58.0) | **Aphasia onset (days)** | 941 (24) |  | 914 | 61 [7, 487] |
| **Ethnicity** | 94 (4) | Black  Caucasian | 5  89 | (5.3)  (94.7) | **Stroke Type** | 771 (17) | Ischaemic  ICH  Subarachnoid Haemorrhage | 685  77  9 | (88.9)  (10.0)  (1.2) |
| **Language** | 959 (24) | English  Dutch | 255  199 | (26.6)  (20.8) | **Stroke Severity** | 298 (4)  216 (4) | NIHSS  mRS | 298  216 | 13 [6, 18]  3 [2, 4] |
|  |  | German  Swedish Italian  Greek | 182  125  38  44 | (19.0)  (13.0)  (4.6)  (4.0) | **Living context** |  | Alone  Formal care  Living with others  Mixed | 146  70  473  12 | (20.8)  (10)  (67.5)  (1.7) |
|  |  | Finnish  Portuguese  Arabic  Korean | 36  30  29  21 | (3.8)  (3.1)  (3.02)  (2.2) | **Handedness** | 620 (17) | Ambidextrous  Left  Right | 7  21  592 | (1.1)  (3.4)  (95.5) |

**Key:** IQR Interquartile range; n (%) or median (IQR); NIHSS = National Institutes of Health Stroke Scale; mRS Modified Rankin Scale

### Supplementary Material H: Younger (≤65 years) and Older (>65 years) subgroups by SLT frequency, intensity and dosage and language outcome

#### (a) SLT frequency and overall language ability (WAB-AQ 0-100)

#### (b) SLT frequency and auditory comprehension (TT-AAT 0-50)

#### (c) SLT frequency and functional communication (AAT-SSC 0-5)


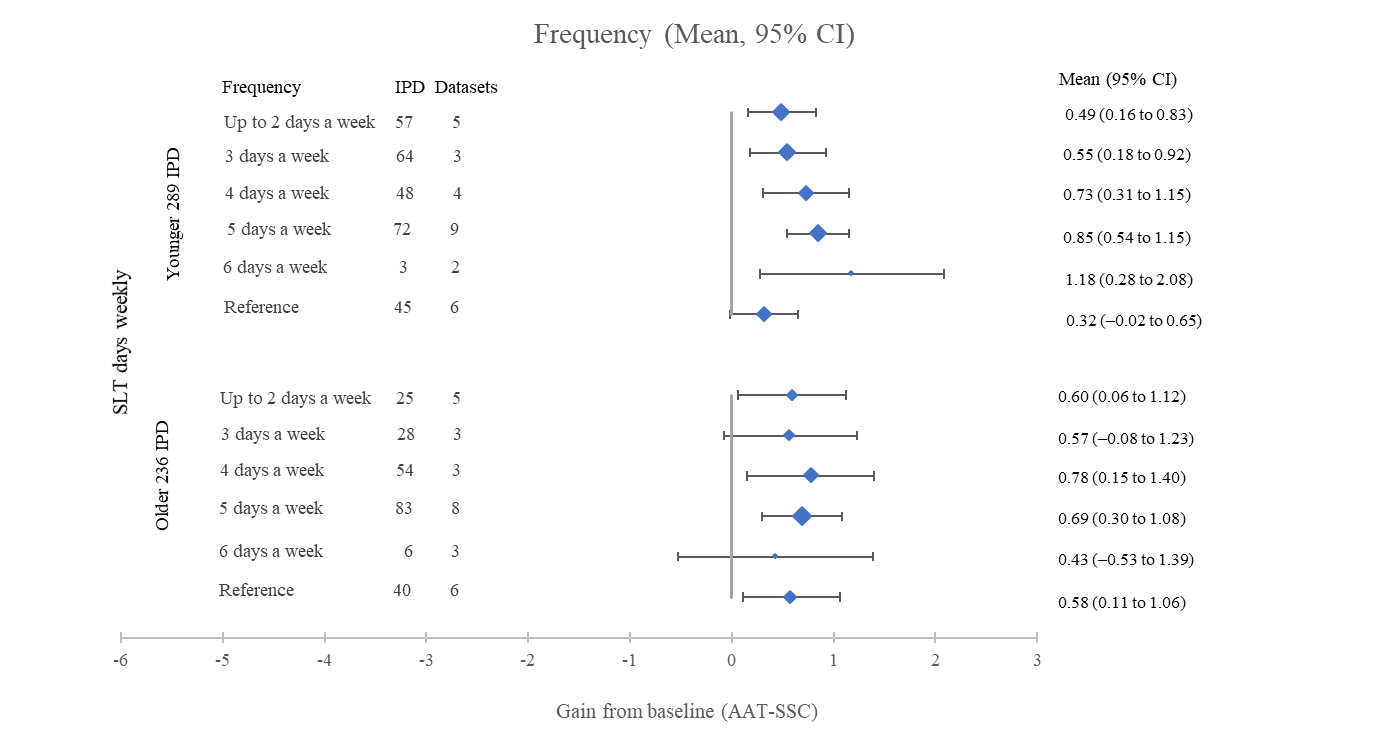


#### (d) SLT intensity and overall language ability (WAB-AQ 0-100)

#### (e) SLT intensity and auditory comprehension (TT-AAT 0-50)

#### (f) SLT intensity and functional communication (AAT-SSC 0-5)

#### (g) SLT dosage and overall language ability (WAB-AQ 0-100)

#### (h) SLT dosage and auditory comprehension (TT-AAT 0-50)

#### (i) SLT dosage and functional communication AAT-SSC 0-5

### Supplementary Materials I: Early (≤ 3 months) and Later (>3 months) after aphasia onset; subgroups by SLT frequency, intensity and dosage and language outcome

#### (a) SLT Frequency and Overall Language (WAB 0-100)

#### (b) Frequency: Auditory Comprehension (TT-AAT 0-50)

#### (c) Frequency: Functional Communication (AAT-SSC 0-5)

#### (d) Intensity: Overall Language (WAB 0-100)

#### (e) Intensity: Auditory Comprehension (TT-AAT 0-50)

#### (f) Intensity: Functional Communication (AAT-SSC 0-5)

#### (g) Dosage: Overall Language (WAB 0-100)

#### (h) Dosage: Auditory Comprehension TT-AAT (0-50)

#### (i) Dosage: Functional Communication (AAT-SSC 0-5)

### Supplementary Material J: Aphasia Severity; Below the median (Moderate-Severe) v above the median (Mild-Moderate): subgroups by SLT frequency, intensity and dosage and language outcome

#### (a) SLT frequency and overall language ability (WAB-AQ 0-100)

#### (b) SLT frequency and auditory comprehension (TT-AAT 0-50)

#### (c) SLT frequency and functional communication (AAT-SSC 0-5)

#### (d) SLT intensity and overall language (WAB-AQ 0-100)

#### (e) SLT intensity and auditory Comprehension (TT-AAT 0-50)

#### (f) SLT intensity and functional communication (AAT-SSC 0-5)

#### (g) SLT dosage and overall language ability (WAB-AQ 0-100)

#### (h) SLT dosage and auditory comprehension TT-AAT (0-50)

#### (i) SLT dosage and functional communication AAT-SSC (0-5)

### Supplementary Material K: Male and Female subgroups by SLT frequency, intensity and dosage and language outcome

#### (a) SLT Frequency and overall language ability (WAB-AQ 0-100)

#### (b) SLT Frequency and auditory comprehension (TT-AAT 0-50)

#### (c) SLT Frequency and functional communication (AAT-SSC 0-5)

#### (d) SLT Intensity and overall language ability (WAB-AQ 0-100)

#### (e) SLT intensity and auditory comprehension (TT-AAT 0-50)

#### (f) SLT intensity and functional communication (AAT-SSC 0-5)

#### (g) SLT dosage and overall language (WAB-AQ 0-100)

#### (h) SLT dosage and auditory comprehension (TT-AAT 0-50)

#### (i) SLT dosage and functional communication (AAT-SSC 0-5)

### Supplementary Material L.

#### Subgroups by language outcome and median SLT frequency, intensity and dosage.

| **Subgroups** | **SLT Frequency (days/week)** | | **SLT Intensity (hours/week)** | | **SLT Dosage**  **(total hours)** | |
| --- | --- | --- | --- | --- | --- | --- |
|  | **Median [IQR]** | **n** | **Median [IQR]** | **n** | **Median [IQR]** | **n** |
| **Overall-Language** | | | | | | |
| Moderate-severe | 4.7 [1-5] | 319 | 3 [2-5] | 274 | 9.3 [4.5-17.2] | 317 |
| Mild-moderate | 4 [1.3-5] | 163 | 3.8 [2-10] | 156 | 14.0 [4.5-16] | 163 |
| Female | 4 [1-5] | 206 | 3 [1.5-5] | 180 | 9.3 [3.6-16] | 205 |
| Male | 4 [1.5-5] | 276 | 3.8 [2-6.8] | 250 | 11.3 [5-175] | 275 |
| <65 years | 4 [2-5] | 277 | 3.8 [2-10] | 256 | 12.5 [5-16] | 276 |
| ≥65 years | 4 [1-5] | 205 | 3 [0-3.8] | 174 | 9.5 [0-19.7] | 205 |
| Early SLT [≤3 mths] | 4 [1-5] | 324 | 2.5 [1-3.8] | 272 | 9.7 [3.8-20] | 322 |
| Late SLT [>3 mths] | 4 [2-5] | 158 | 6.8 [3-10] | 158 | 14 [4.5-15] | 158 |
| **Auditory comprehension** | | | | | | |
| Moderate-severe | 3.3 [2.3-5] | 404 | 3.8 [2.3-7.3] | 404 | 17 [5.5-50] | 404 |
| Mild-moderate | 3.3 [2-5] | 146 | 3.5 [2-10] | 146 | 19.3 [7.5-50] | 146 |
| Female | 3.3 [2.3-5] | 211 | 3.5 [2.3-7.3] | 211 | 25 [7.5-52] | 211 |
| Male | 3.3 [2.3-5] | 329 | 2.8 [2.3-10] | 329 | 16 [6.8-50] | 329 |
| <65 years | 4 [2.1-5] | 360 | 4.5 [2.3-10] | 360 | 15 [5.4-50] | 360 |
| ≥65 years | 3.3 [2.3-5] | 180 | 3.5 [2.3-5] | 180 | 48 [7.5-52] | 180 |
| Early SLT [≤3 mths] | 3.3 [3-5] | 236 | 3.5 [2-5] | 236 | 30 [7.5-52] | 236 |
| Late SLT [>3 mths] | 4 [2.3-5] | 304 | 6 [2.3-10] | 304 | 15 [6.8-45] | 304 |
| **Functional communication** | | | | | | |
| Moderate-severe | 3.3 [1-5] | 280 | 3.5 [2-4.5], | 245 | 30 [3.8-50] | 278 |
| Mild-moderate | 3.0 [2-4.9] | 251 | 3.5 [2.3-3.8] | 234 | 24 [6-50] | 251 |
| Female | 3.3 [1-5] | 237 | 3.5 [2-3.8] | 211 | 30 [4.5-52] | 236 |
| Male | 3 [1-5] | 294 | 3.5 [2.3-4.5] | 268 | 22.6 [4.5-50] | 293 |
| <65 years | 3 [1-4] | 291 | 3.5 [2-5] | 270 | 30 [4.5-50] | 290 |
| ≥65 years | 3.3 [1.1-5] | 240 | 3.5 [2.3-3.8] | 209 | 24.0 [6.8-52] | 239 |
| Early SLT [≤3 mths] | 3.3 [1-5] | 300 | 3.5 [2-3.8] | 248 | 24.0 [7.5-52] | 298 |
| Late SLT [>3 mths] | 2.3 [2-4] | 226 | 3.0 [2.3-6] | 226 | 30 [4.5-40] | 226 |

### Supplementary Materials M:

#### Base Models by age, time since aphasia onset, aphasia severity and sex.

| Base Model | | | RCT | | | | | | | | | IPD | | | Estimate of means  (CI 95%) | | | | RCT | | | | | | | | | IPD | | | | | Estimate of means  (CI 95%) | | |
| --- | --- | --- | --- | --- | --- | --- | --- | --- | --- | --- | --- | --- | --- | --- | --- | --- | --- | --- | --- | --- | --- | --- | --- | --- | --- | --- | --- | --- | --- | --- | --- | --- | --- | --- | --- |
| **Younger (≤ 65 years) versus Older (>65 years) subgroups** | | | | | | | | | | | | | | | | | | | | | | | | | | | | | | | | | | | |
| **Overall language ability on WAB-AQ; range 0-100** | | | | | | | | | | | | | | | | | | | | | | | | | | | | | | | | | | | |
| Female | | | 11 | | | | | | 97 | | | | | | | 13.97 (7.98, 19.97) | | | | 9 | | | | | | | 109 | | | | 13.86 (6.92, 20.79) | | | | |
| Male | | | 11 | | | | | | 180 | | | | | | | 12.21 (6.53, 17.89) | | | | 10 | | | | | | | 96 | | | | 11.74 (5.02, 18.45) | | | | |
| 0 to 1 month | | | 8 | | | | | | 97 | | | | | | | 17.89 (11.84, 23.95) | | | | 8 | | | | | | | 163 | | | | 22.36 (16.78, 27.95) | | | | |
| >1 to 3 months | | | 6 | | | | | | 45 | | | | | | | 16.44 (9.77, 23.11) | | | | 5 | | | | | | | 19 | | | | 11.71 (2.60, 20.82) | | | | |
| >3 to 6 months | | | 3 | | | | | | 10 | | | | | | | 9.52 (-1.33, 20.36) | | | | 1 | | | | | | | 6 | | | | 11.18 (-5.05, 27.41) | | | | |
| 6+ months | | | 4 | | | | | | 125 | | | | | | | 8.53 (-0.33, 17.39) | | | | 2 | | | | | | | 17 | | | | 5.94 (-6.00, 17.88) | | | | |
| **Auditory Comprehension on TT-AAT;** **range 0-50** | | | | | | | | | | | | | | | | | | | | | | | | | | | | | | | | | | | |
| Female | | | 16 | | | | | | 135 | | | | | | | 4.63 (1.73, 7.53) | | | | 13 | | | | | | | 76 | | | | 4.50 (1.09, 7.92) | | | | |
| Male | | | 16 | | | | | | 225 | | | | | | | 3.95 (1.20, 6.69) | | | | 15 | | | | | | | 104 | | | | 3.22 (0.05, 6.38) | | | | |
| 0 to 1 month | | | 6 | | | | | | 62 | | | | | | | 7.66 (3.49, 11.82) | | | | 6 | | | | | | | 77 | | | | 4.77 (0.70, 8.83) | | | | |
| >1 to 3 months | | | 9 | | | | | | 70 | | | | | | | 3.91 (0.54, 7.27) | | | | 8 | | | | | | | 27 | | | | 6.63 (2.22, 11.05) | | | | |
| >3 to 6 months | | | 5 | | | | | | 37 | | | | | | | 3.80 (-1.48, 9.08) | | | | 3 | | | | | | | 24 | | | | 2.54 (-3.95, 9.03) | | | | |
| 6+ months | | | 9 | | | | | | 191 | | | | | | | 1.79 (-1.65, 5.23) | | | | 7 | | | | | | | 52 | | | | 1.50 (-2.48, 5.48) | | | | |
| **Naming on BNT; range 0-60** | | | | | | | | | | | | | | | | | | | | | | | | | | | | | | | | | | | |
| Female | | | 13 | | | | 87 | | | | | | | | | 9.85 (5.30, 14.40) | | | | 11 | | | | | | | | | 78 | | | 4.01 **(**-0.96**,** 8.98**)** | | | |
| Male | | | 13 | | | | 140 | | | | | | | | | 7.21 (2.87, 11.54) | | | | 12 | | | | | | | | | 80 | | | 7.44 (2.61, 12.27) | | | |
| 0 to 1 month | | | 5 | | | | 55 | | | | | | | | | 14.61 (8.33, 20.88) | | | | 5 | | | | | | | | | 74 | | | 11.56 (5.29, 17.83) | | | |
| >1 to 3 months | | | 8 | | | | 65 | | | | | | | | | 9.15 (4.16, 14.14) | | | | 7 | | | | | | | | | 28 | | | 6.68 (0.26, 13.10) | | | |
| >3 to 6 months | | | 5 | | | | 44 | | | | | | | | | 5.21 (-1.06, 11.47) | | | | 3 | | | | | | | | | 26 | | | 1.91 (-6.48, 10.31) | | | |
| 6+ months | | | 7 | | | | 63 | | | | | | | | | 5.15 (-0.22, 10.52) | | | | 5 | | | | | | | | | 30 | | | 2.74 (-4.02, 9.50) | | | |
| **Functional Communication on the AAT-SSC; range 0 to 5** | | | | | | | | | | | | | | | | | | | | | | | | | | | | | | | | | | | |
| Female | | | 14 | | | | | 109 | | | | | | 0.79 (0.52, 1.06) | | | | 14 | | | | | 127 | | | | | | | | | | | | 0.69 (0.41, 0.97) |
| Male | | | 14 | | | | | 183 | | | | | | 0.55 (0.29, 0.81) | | | | 13 | | | | | 113 | | | | | | | | | | | | 0.57 (0.30, 0.83) |
| 0 to 1 month | | | 6 | | | | | 78 | | | | | | 1.19 (0.83, 1.55) | | | | 6 | | | | | 154 | | | | | | | | | | | | 1.11 (0.82, 1.39) |
| >1 to 3 months | | | 5 | | | | | 46 | | | | | | 0.86 (0.50, 1.22) | | | | 5 | | | | | 22 | | | | | | | | | | | | 0.87(0.37, 1.36) |
| >3 to 6 months | | | 3 | | | | | 38 | | | | | | 0.27 (-0.20, 0.75) | | | | 3 | | | | | 24 | | | | | | | | | | | | 0.28 (-0.27, 0.84) |
| 6+ months | | | 7 | | | | | 130 | | | | | | 0.36 (0.05, 0.67) | | | | 7 | | | | | 40 | | | | | | | | | | | | 0.25 (-0.13, 0.63) |
| **Early SLT (≤ 3 months) versus Late SLT (> 3 months since onset) subgroups** | | | | | | | | | | | | | | | | | | | | | | | | | | | | | | | | | | | |
| **Overall language ability on WAB-AQ; range 0-100** | | | | | | | | | | | | | | | | | | | | | | | | | | | | | | | | | | | |
| Female | | | | | 10 | | | | | | 150 | | 21.54 (15.70, 27.38) | | | | | | | | 4 | | | | | | | | | 56 | | | | | 13.86 (6.92, 20.79) |
| Male | | | | | 10 | | | | | | 174 | | 17.99 (12.35, 23.64) | | | | | | | | 4 | | | | | | | | | 102 | | | | | 11.74 (5.02, 18.45) |
| 55+ years | | | | | 10 | | | | | | 55 | | 21.13 (14.30, 27.96) | | | | | | | | 4 | | | | | | | | | 81 | | | | | 6.98 (4.30, 9.65) |
| 56 to 65 years | | | | | 10 | | | | | | 87 | | 18.98 (12.73, 25.23) | | | | | | | | 3 | | | | | | | | | 54 | | | | | 4.13 (1.06, 7.19) |
| 66 to 75 years | | | | | 9 | | | | | | 76 | | 18.60 (12.15, 25.05) | | | | | | | | 2 | | | | | | | | | 20 | | | | | 2.70 (-1.67, 7.06) |
| 75+ years | | | | | 6 | | | | | | 106 | | 20.35 (13.86, 26.85) | | | | | | | | 1 | | | | | | | | | 3 | | | | | 5.33 (-5.08, 15.73) |
| **Auditory Comprehension on TT-AAT;** **range 0-50** | | | | | | | | | | | | | | | | | | | | | | | | | | | | | | | | | | | |
| Female | | | | 11 | | | | | | 112 | | | 4.00 (-0.78, 8.79) | | | | | | | | 10 | | | | | | | | | 99 | | | | | 2.58 (0.50, 4.66) |
| Male | | | | 11 | | | | | | 124 | | | 3.36 (-1.41, 8.12) | | | | | | | | 11 | | | | | | | | | 205 | | | | | 1.93 (0.11, 3.75) |
| 55+ years | | | | 10 | | | | | | 53 | | | 7.12 (1.93, 12.30) | | | | | | | | 9 | | | | | | | | | 125 | | | | | 4.03 (2.06, 6.01) |
| 56 to 65 years | | | | 11 | | | | | | 79 | | | 2.51 (-2.37, 7.39) | | | | | | | | 9 | | | | | | | | | 103 | | | | | 1.80 (-0.27, 3.86) |
| 66 to 75 years | | | | 9 | | | | | | 59 | | | 4.12 (-1.05, 9.29) | | | | | | | | 9 | | | | | | | | | 57 | | | | | 1.37 (-0.95, 3.70) |
| 75+ years | | | | 7 | | | | | | 45 | | | 0.97 (-4.41, 6.35) | | | | | | | | 6 | | | | | | | | | 19 | | | | | 1.82 (-1.70, 5.33) |
| **Naming on BNT; range 0-60** | | | | | | | | | | | | | | | | | | | | | | | | | | | | | | | | | | | |
| Female | | | | | 9 | | | | | 104 | | | 9.29 (1.49, 17.09) | | | | | | | 8 | | | | | | | | | | 61 | | | | | 2.20 (0.58, 3.82) |
| Male | | | | | 9 | | | | | 118 | | | 9.35 (1.60, 17.10) | | | | | | | 9 | | | | | | | | | | 102 | | | | | 1.26 (-0.20, 2.73) |
| 55+ years | | | | | 8 | | | | | 44 | | | 13.30 (4.93, 21.66) | | | | | | | 8 | | | | | | | | | | 59 | | | | | 3.28 (1.68, 4.88) |
| 56 to 65 years | | | | | 9 | | | | | 76 | | | 9.20 (1.33, 17.07) | | | | | | | 7 | | | | | | | | | | 48 | | | | | 2.23 (0.41, 4.05) |
| 66 to 75 years | | | | | 7 | | | | | 58 | | | 8.18 (-0.06, 16.41) | | | | | | | 7 | | | | | | | | | | 39 | | | | | 1.17 (-0.71, 3.05) |
| 75+ years | | | | | 6 | | | | | 44 | | | 6.60 (-1.87, 15.08) | | | | | | | 6 | | | | | | | | | | 17 | | | | | 0.24 (-2.44, -2.92) |
| **Functional Communication on AAT-SSC; range 0 to 5** | | | | | | | | | | | | | | | | | | | | | | | | | | | | | | | | | | | |
| Female | 8 | | | | | | | | | 151 | | | | | | 1.12 (0.77, 1.48) | | | | 8 | | | | | | | | | | 86 | | | | | 0.37 (-0.06, 0.69) |
| Male | 8 | | | | | | | | | 149 | | | | | | 0.93 (0.57, 1.29) | | | | 9 | | | | | | | | | | 147 | | | | | 0.14 (-0.23, 0.51) |
| 55+ years | 7 | | | | | | | | | 53 | | | | | | 1.15 (0.73, 1.58) | | | | 8 | | | | | | | | | | 94 | | | | | 0.29 (-0.09, 0.67) |
| 56 to 65 years | 8 | | | | | | | | | 71 | | | | | | 1.05 (0.65, 1.44) | | | | 7 | | | | | | | | | | 74 | | | | | 0.25 (-0.14, 0.64) |
| 66 to 75 years | 7 | | | | | | | | | 79 | | | | | | 0.89 (0.49, 1.28) | | | | 9 | | | | | | | | | | 43 | | | | | 0.14 (-0.26, 0.54) |
| 75+ years | 7 | | | | | | | | | 97 | | | | | | 1.02 (0.63, 1.42) | | | | 6 | | | | | | | | | | 22 | | | | | 0.23 (-0.23, 0.69) |
| **Moderate-severe aphasia versus mild-moderate aphasia subgroups by language outcome** | | | | | | | | | | | | | | | | | | | | | | | | | | | | | | | | | | |  |
| **Overall language ability on the WAB-AQ; range 0-100:**  **Moderate-severe (below the median: <64.9 (n=319))** | | | | | | | | | | | | | | | | | | | | **Mild-moderate (≥ 64.9 (n=163))** | | | | | | | | | | | | | | | |
| Female | | | | | | 11 | | | | 138 | | | | | | 17.81 (11.13, 24.50) | | | | 9 | | 68 | | | | | | | | | | | | | 5.95 (1.36, 10.55) |
| Male | | | | | | 11 | | | | 181 | | | | | | 14.66 (8.17, 21.15) | | | | 10 | | 95 | | | | | | | | | | | | | 6.87 (2.34, 11.40) |
| 55+years | | | | | | 11 | | | | 76 | | | | | | 19.60 (12.49, 26.71) | | | | 9 | | 60 | | | | | | | | | | | | | 7.50 (2.8, 12.19) |
| 56 to 65 years | | | | | | 11 | | | | 91 | | | | | | 13.99 (6.93, 21.06) | | | | 6 | | 50 | | | | | | | | | | | | | 8.36 (3.61, 13.12) |
| 66 to 75 years | | | | | | 10 | | | | 71 | | | | | | 14.23 (6.94, 21.53) | | | | 7 | | 25 | | | | | | | | | | | | | 5.66 (0.52, 10.80) |
| 75+years | | | | | | 7 | | | | 81 | | | | | | 17.13 (9.33, 24.93) | | | | 6 | | 28 | | | | | | | | | | | | | 4.11 (-1.04, 9.26) |
| 0 to 1 month | | | | | | 7 | | | | 199 | | | | | | 24.10 (17.62, 30.59) | | | | 7 | | 61 | | | | | | | | | | | | | 10.95 (6.18, 15.73) |
| >1 to 3 months | | | | | | 6 | | | | 46 | | | | | | 22.15 (14.43, 29.86) | | | | 3 | | 18 | | | | | | | | | | | | | 5.21 (-0.68, 11.10) |
| >3 to 6 months | | | | | | 3 | | | | 10 | | | | | | 9.25 (-4.43, 22.93) | | | | 2 | | 6 | | | | | | | | | | | | | 5.54 (-1.71, 12.79) |
| 6+ months | | | | | | 4 | | | | 64 | | | | | | 9.45 (-2.25, 21.16) | | | | 4 | | 78 | | | | | | | | | | | | | 3.94 (-2.19, 10.06) |
| **Auditory Comprehension on the TT-AAT;** **range 0-50**  **Moderate-severe (below median <35 (n=395))** | | | | | | | | | | | | | | | | | | | | **Mild-moderate (≥ 35 (n=145))** | | | | | | | | | | | | | | | |
| Female | | | | | | 16 | | | | 155 | | | | | | 5.40 (2.11, 8.69) | | | | 12 | | 56 | | | | | | | | | | | | | 0.65 (-3.83, 5.13) |
| Male | | | | | | 16 | | | | 240 | | | | | | 5.59 (2.37, 8.83) | | | | 12 | | 89 | | | | | | | | | | | | | -1.24 (-5.61, 3.13) |
| 55+years | | | | | | 15 | | | | 133 | | | | | | 8.22 (4.90, 11.56) | | | | 9 | | 45 | | | | | | | | | | | | | 1.50 (-3.24, 6.22) |
| 56 to 65 years | | | | | | 16 | | | | 132 | | | | | | 5.33 (1.99, 8.68) | | | | 13 | | 50 | | | | | | | | | | | | | -1.24 (-5.75, 3.28) |
| 66 to 75 years | | | | | | 15 | | | | 88 | | | | | | 5.21 (1.70, 8.73) | | | | 9 | | 28 | | | | | | | | | | | | | -0.46 (-5.28, 4.37) |
| 75+years | | | | | | 11 | | | | 42 | | | | | | 3.20 (-0.80, 7.20) | | | | 8 | | 22 | | | | | | | | | | | | | -0.98 (-5.94, 3.97) |
| 0 to 1 month | | | | | | 6 | | | | 88 | | | | | | 8.63 (4.51, 12.75) | | | | 5 | | 51 | | | | | | | | | | | | | -0.12 (-5.82, 5.59) |
| >1 to 3 months | | | | | | 9 | | | | 80 | | | | | | 6.72 (3.11, 10.32) | | | | 5 | | 17 | | | | | | | | | | | | | 0.21 (-5.52, 5.93) |
| >3 to 6 months | | | | | | 4 | | | | 50 | | | | | | 4.37 (-1.43, 10.17) | | | | 4 | | 11 | | | | | | | | | | | | | -1.77 (-8.08, 4.54) |
| 6+ months | | | | | | 9 | | | | 177 | | | | | | 2.25 (-1.49, 5.99) | | | | 8 | | 66 | | | | | | | | | | | | | 0.50 (-4.79, 5.79) |
| **Naming on BNT; range 0-60;**  **Moderate-severe (below the median <23 (n=304))** | | | | | | | | | | | | | | | | | | | | **Mild-moderate (≥ 23 (n=81))** | | | | | | | | | | | | | | | |
| Female | | | | | | 12 | | | | 130 | | | | | | 6.84 (1.98, 11.71) | | | | 8 | | 35 | | | | | | | | | | | | | 7.59 (3.79, 11.38) |
| Male | | | | | | 13 | | | | 174 | | | | | | 6.99 (2.27, 11.70) | | | | 8 | | 46 | | | | | | | | | | | | | 8.36 (4.57, 12.14) |
| 55+years | | | | | | 12 | | | | 79 | | | | | | 9.53 (4.50, 14.56) | | | | 7 | | 24 | | | | | | | | | | | | | 8.79 (4.59, 13.00) |
| 56 to 65 years | | | | | | 12 | | | | 97 | | | | | | 7.39 (2.38, 12.39) | | | | 7 | | 27 | | | | | | | | | | | | | 8.13 (4.05, 12.21) |
| 66 to 75 years | | | | | | 12 | | | | 84 | | | | | | 6.76 (1.67, 11.84) | | | | 6 | | 13 | | | | | | | | | | | | | 6.74 (1.66, 11.82) |
| 75+years | | | | | | 9 | | | | 44 | | | | | | 3.98 (-1.59, 9.56) | | | | 6 | | 17 | | | | | | | | | | | | | 8.22 (3.43, 13.02) |
| 0 to 1 month | | | | | | 5 | | | | 99 | | | | | | 12.22 (6.35, 18.09) | | | | 3 | | 30 | | | | | | | | | | | | | 11.61 (6.25, 16.97) |
| >1 to 3 months | | | | | | 8 | | | | 78 | | | | | | 7.99 (2.67, 13.30) | | | | 3 | | 15 | | | | | | | | | | | | | 9.37 (3.76, 14.97) |
| >3 to 6 months | | | | | | 6 | | | | 62 | | | | | | 3.56 (-3.10, 10.21) | | | | 2 | | 8 | | | | | | | | | | | | | 6.83 (0.32, 13.33) |
| 6+ months | | | | | | 7 | | | | 65 | | | | | | 3.89 (-1.81, 9.59) | | | | 5 | | 28 | | | | | | | | | | | | | 4.08 (-0.37, 8.54) |
| **Functional Communication on the AAT-SSC; range 0 to 5**  **Moderate-severe (below the median: <3.7 (n=433))** | | | | | | | | | | | | | | | | | | | | **Mild-moderate (≥3.7 (n=100))** | | | | | | | | | | | | | | | |
| Female | | | | | | 14 | | | | 189 | | | | | | 0.93 (0.65, 1.22) | | | | 9 | | 48 | | | | | | | | | | | | | -0.01 (-0.24, 0.21) |
| Male | | | | | | 14 | | | | 244 | | | | | | 0.70 (0.42, 0.98) | | | | 10 | | 52 | | | | | | | | | | | | | 0.03 (-0.16, 0.23) |
| 55+years | | | | | | 13 | | | | 118 | | | | | | 0.88 (0.58, 1.18) | | | | 7 | | 29 | | | | | | | | | | | | | 0.03 (-0.22, 0.29) |
| 56 to 65 years | | | | | | 13 | | | | 120 | | | | | | 0.90 (0.60, 1.21) | | | | 9 | | 25 | | | | | | | | | | | | | -0.19 (-0.42, 0.04) |
| 66 to 75 years | | | | | | 14 | | | | 101 | | | | | | 0.66 (0.35, 0.98) | | | | 7 | | 21 | | | | | | | | | | | | | 0.11 (-0.15, 0.37) |
| 75+years | | | | | | 12 | | | | 94 | | | | | | 0.82 (0.49, 1.14) | | | | 6 | | 25 | | | | | | | | | | | | | 0.09 (-0.17, 0.36) |
| 0 to 1 month | | | | | | 6 | | | | 183 | | | | | | 1.33 (0.95, 1.70) | | | | 6 | | 49 | | | | | | | | | | | | | 0.33 (0.19, 0.47) |
| >1 to 3 months | | | | | | 5 | | | | 62 | | | | | | 0.96 (0.58, 1.33) | | | | 3 | | 6 | | | | | | | | | | | | | 0.32 ( -0.04, 0.68) |
| >3 to 6 months | | | | | | 4 | | | | 61 | | | | | | 0.55 (0.06, 1.03) | | | | 2 | | 2 | | | | | | | | | | | | | -0.56 (-1.19, 0.08) |
| 6+ months | | | | | | 7 | | | | 127 | | | | | | 0.44 (0.10, 0.77) | | | | 4 | | 43 | | | | | | | | | | | | | -0.05 (-0.21, 0.11) |
| **Male (IPD 329) versus female (IPD 211) subgroups** | | | | | | | | | | | | | | | | | | | | | | | | | | | | | | | | | | | |
| **Overall language ability WAB-AQ range 0-100** | | | | | | | | | | | | | | | | | | | | | | | | | | | | | | | | | | | |
| 55 years | | | 11 | | | | | | | | | 81 | | | | | 14.08 (7.70, 20.46) | | | 10 | | | | 55 | | | | | | | | | | 16.28 (8.84, 23.72) | |
| 56 to 65 years | | | 11 | | | | | | | | | 99 | | | | | 10.92 (4.70, 17.14) | | | 10 | | | | 42 | | | | | | | | | | 13.68 (6.01, 21.36) | |
| 66 to 75 years | | | 10 | | | | | | | | | 55 | | | | | 8.86 (2.03, 15.68) | | | 9 | | | | 41 | | | | | | | | | | 15.07 (7.15, 22.99) | |
| 75+ years | | | 7 | | | | | | | | | 41 | | | | | 12.17 (4.75, 19.59) | | | 6 | | | | 68 | | | | | | | | | | 16.63 (8.38, 24.89) | |
| 0 to 1 month | | | 8 | | | | | | | | | 133 | | | | | 16.07 (9.99, 22.16) | | | 7 | | | | 127 | | | | | | | | | | 22.86 (16.17, 29.55) | |
| >1 to 3 months | | | 6 | | | | | | | | | 41 | | | | | 14.58 (7.56, 21.59) | | | 5 | | | | 23 | | | | | | | | | | 18.46 (9.78, 27.13) | |
| >3 to 6 months | | | 3 | | | | | | | | | 9 | | | | | 8.29 (-3.37, 19.95) | | | 3 | | | | 7 | | | | | | | | | | 11.15 (-3.24, 25.54) | |
| 6+ months | | | 4 | | | | | | | | | 93 | | | | | 7.09 (-2.10, 16.27) | | | 3 | | | | 49 | | | | | | | | | | 9.20 (-3.52, 21.93) | |
| **Auditory Comprehension on TT-AAT range 0-50** | | | | | | | | | | | | | | | | | | | | | | | | | | | | | | | | | | | |
| 55+ years | | | 15 | | | | | | | | | 103 | | | | | 6.05 (2.72, 9.39) | | | 13 | | | | 75 | | | | | | | | | | | 6.27 (2.95, 9.59) |
| 56 to 65 years | | | 16 | | | | | | | | | 122 | | | | | 2.36 (-0.90, 5.61) | | | 15 | | | | 60 | | | | | | | | | | | 3.98 (0.58, 7.38) |
| 66 to 75 years | | | 15 | | | | | | | | | 74 | | | | | 2.47 (-1.00, 5.94) | | | 13 | | | | 42 | | | | | | | | | | | 5.87 (2.15, 9.59) |
| 75+ years | | | 10 | | | | | | | | | 30 | | | | | 1.13 (-3.16, 5.42) | | | 8 | | | | 34 | | | | | | | | | | | 2.93 (-1.06, 6.91) |
| 0 to 1 month | | | 6 | | | | | | | | | 72 | | | | | 6.00 (1.58, 10.41) | | | 6 | | | | 67 | | | | | | | | | | | 5.53 (1.55, 9.51) |
| >1 to 3 months | | | 9 | | | | | | | | | 52 | | | | | 4.19 (0.30, 8.09) | | | 9 | | | | 45 | | | | | | | | | | | 5.13 (1.36, 8.90) |
| >3 to 6 months | | | 6 | | | | | | | | | 39 | | | | | 1.43 (-4.12, 6.97) | | | 3 | | | | 22 | | | | | | | | | | | 5.70 (-0.84, 12.24) |
| 6+ months | | | 8 | | | | | | | | | 166 | | | | | 0.39 (-3.34, 4.12) | | | 8 | | | | 77 | | | | | | | | | | | 2.69 (-1.41, 6.80) |
| **Naming on BNT range 0-60** | | | | | | | | | | | | | | | | | | | | | | | | | | | | | | | | | | | |
| 55+ years | | | 12 | | | | | | | | | 55 | | | | | 7.88 (3.32, 12.44) | | | 11 | | | | | 48 | | | | | | | | | | 10.35 (4.35, 16.34) |
| 56 to 65 years | | | 13 | | | | | | | | | 85 | | | | | 6.42 (2.13, 10.71) | | | 13 | | | | | 39 | | | | | | | | | | 9.44 (3.33, 15.54) |
| 66 to 75 years | | | 12 | | | | | | | | | 56 | | | | | 6.68 (2.10, 11.25) | | | 11 | | | | | 41 | | | | | | | | | | 6.69 (0.46, 12.91) |
| 75+ years | | | 8 | | | | | | | | | 24 | | | | | 7.72 (2.03, 13.42) | | | 10 | | | | | 37 | | | | | | | | | | 3.59 (-2.70, 9.89) |
| 0 to 1 month | | | 5 | | | | | | | | | 66 | | | | | 15.93 (10.23, 21.63) | | | 5 | | | | | 63 | | | | | | | | | | 8.54 (1.45, 15.63) |
| >1 to 3 months | | | 7 | | | | | | | | | 52 | | | | | 7.62 (2.75, 12.52) | | | 8 | | | | | 41 | | | | | | | | | | 8.86 (2.34, 15.39) |
| >3 to 6 months | | | 6 | | | | | | | | | 44 | | | | | 2.10 (-4.03, 8.24) | | | 4 | | | | | 26 | | | | | | | | | | 7.46 (-0.85, 15.76) |
| 6+ months | | | 7 | | | | | | | | | 58 | | | | | 3.05 (-2.08, 8.18) | | | 6 | | | | | 35 | | | | | | | | | | 5.21 (-2.15, 12.57) |
| **Functional Communication AAT-SSC* range 0 to 5 (Male n=296; Female n=237)** | | | | | | | | | | | | | | | | | | | | | | | | | | | | | | | | | | | |
| 55+ years | | 13 | | | | | | | | | | 77 | | | | | 0.49 (0.25, 0.72) | | | 12 | | | | | | 70 | | | | | | | | | 1.01 (0.56, 1.46) |
| 56 to 65 years | | 13 | | | | | | | | | | 106 | | | | | 0.56 (0.35, 0.76) | | | 12 | | | | | | 39 | | | | | | | | | 0.81 (0.33, 1.30) |
| 66 to 75 years | | 13 | | | | | | | | | | 70 | | | | | 0.44 (0.20, 0.67) | | | 14 | | | | | | 52 | | | | | | | | | 0.74 (0.27, 1.21) |
| 75+ years | | 10 | | | | | | | | | | 43 | | | | | 0.60 (0.30, 0.91) | | | 12 | | | | | | 76 | | | | | | | | | 0.77 (0.31, 1.23) |
| 0 to 1 month | | 6 | | | | | | | | | | 117 | | | | | 1.15 (0.93, 1.38) | | | 6 | | | | | | 115 | | | | | | | | | 1.07 (0.54, 1.60) |
| >1 to 3 months | | 5 | | | | | | | | | | 32 | | | | | 0.65 (0.31, 0.99) | | | 5 | | | | | | 36 | | | | | | | | | 1.05 (0.51, 1.59) |
| >3 to 6 months | | 4 | | | | | | | | | | 40 | | | | | 0.11 (-0.25, 0.47) | | | 2 | | | | | | 23 | | | | | | | | | 0.80 (0.01, 1.59) |
| 6+ months | | 7 | | | | | | | | | | 107 | | | | | 0.17 (-0.07, 0.40) | | | 7 | | | | | | 63 | | | | | | | | | 0.41 (-0.10, 0.93) |

Key: RCT randomised controlled trial; IPD individual participant data;

### Supplementary Material N:

#### Risk of Bias by included trial dataset*


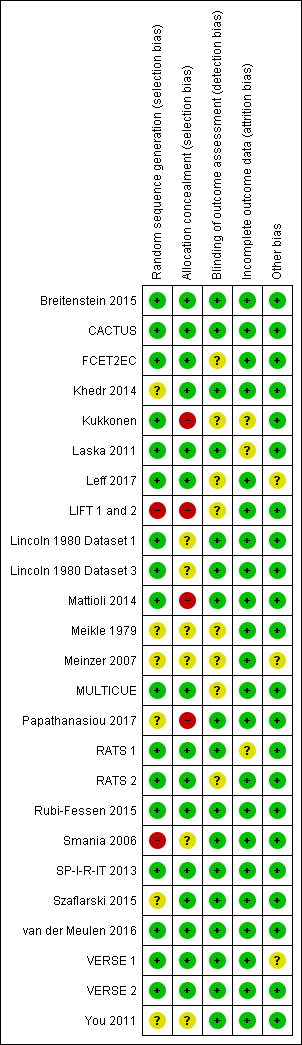


#### Originally reported in:

#### The RELEASE Collaborators. Impact of frequency, intensity and dosage of language therapy for people with aphasia after stroke: a systematic review and individual participant data network meta-analysis . Stroke 2021; [/doi.org/10.1161/STROKEAHA.121.035216](https://doi.org/10.1161/STROKEAHA.121.035216)

#### Additional Results - Heterogeneity

Collaborators confirmed that included interventions were SLT, and these were categorised through consensus (19). Therapy regimen, delivery, and content differences were examined in an a-priori analysis and reported elsewhere (20). Our analyses revealed 10-25% relative variance in most instances. Risk of primary and meta-biases was moderate to low; random sequence generation (17 RCTs; 68%) and concealment of allocation was adequate (15 RCTs; 60%); 68% (17 RCTs) reported outcome assessor blinding(20). Participants were retained, or dropouts and non-adherence were fully reported. Most groups were comparable by age, sex, time since stroke, and aphasia severity (where available) at baseline (20). We found no evidence in sensitivity analyses that fixed versus random-effects model, historic dataset exclusion, publication date, or outcome measure choice would have altered the findings (20).

### Supplementary Materials O

#### Author contributions

Author contributions are listed by contribution, followed by the order of authors as they appear in the authorship list. First, middle and last name initials are used. Where duplicates exist, abbreviations are used: MA1 Myzoon Ali; MA2 Mashiro Abo; CB1 Caitlin Brandenburg; CB2 Caterina Breitenstein.

MCB conceived, designed, and led the study, assessed the risk of bias, drafted and finalised the manuscript. MB, KVB, LRW screened records, abstracts, and full titles, extracted data, checked data extraction and risk of bias. KVB, LRW Retrieved papers. MA2, FB, AB, CB1, CB2, SB, DAC, TBC, MdiP-B, PE, JF, FLG, MG, BG, EG, KH, JH, SH, PJ, EJ, LMTJ, MK, EYK, EMK, AP-HK, TK, ML, MALP, ACL, BL, APL, RRL, AL, BMacW, RSM, FM, IM, MM, RN, EN, N-JP, RP, IP, BFP, IPM, CP, TPJ, ER, MLR, CR, IR-F, MBR, CS, BS, JPS, SAT, MvdS-K, IvdM, EV-B, LW, HHW contributed IPD primary data. LJW and MA analysed the data. NH Advised on the statistical analysis SH Co-ordinated and Facilitated Patient and Public Involvement in the study. All authors were involved in the interpretation of the results, reviewing and approving of this manuscript.

#### Declaration of interests

MCB reports grants from the Chief Scientist Office, the Scottish Government Health and Social Care Directorates, the European Union Cooperation in Science and Technology (COST)-funded Collaboration of Aphasia Scientists [IS1208, [www.aphasiatrials.org](https://www.aphasiatrials.org/) (accessed 5 June 2020)] and The Tavistock Trust for Aphasia, during the conduct of the study, and is a member of the Royal College of Speech and Language Therapists. Audrey Bowen reports that data from her research is included in the analyses in the REhabilitation and recovery of peopLE with Aphasia after StrokE (RELEASE) report. Her post at the University of Manchester is partly funded by research grants and personal awards from the National Institute for Health Research (NIHR) and the Stroke Association. Caterina Breitenstein reports grants from the German Federal Ministry of Education and Research during the conduct of the study. Erin Godecke reports Western Australian State Health Research Advisory Council Research Translation Project grants RSD-02720; 2008/9, during the conduct of the study. Neil Hawkins reports grants from NIHR during the conduct of the study. Katerina Hilari reports grants from the Stroke Association, from the European Social Fund and Greek National Strategic Reference Framework, and from The Tavistock Trust for Aphasia outside the submitted work. Petra Jaecks reports a Ph.D. grant from Weidmüller Stiftung. Anthony Pak-Hin Kong reports funding from the National Institutes of Health (NIH). Brian MacWhinney reports grants from the National Institutes of Health (NIH). Rebecca Marshall reports grants from the National Institute of Deafness and Other Communication Disorders and NIH during the conduct of the study. Rebecca Palmer reports grants from the NIHR senior clinical academic lectureship, from the NIHR Health Technology Assessment programme, and from The Tavistock Trust for Aphasia outside the submitted work. Ilias Papathanasiou reports funding from the European Social Fund and Greek National Strategic Reference Framework. Jerzy Szaflarski reports personal fees from SK Life Sciences (Fair Lawn, NJ, USA), LivaNova Inc. (Houston, TX, USA), Lundbeck (Deerfield, IL, USA), NeuroPace Inc. (Mountain View, CA, USA), Upsher-Smith Laboratories, LLC (Maple Grove, MN, USA). He also reports grants and personal fees from Sage Therapeutics, Inc. (Cambridge, MA, USA) and Union Chimique Belge (UCB) S.A. (Brussels, Belgium), grants from Biogen Inc. (Cambridge, MA, USA) and Eisai Co., Ltd (Tokyo, Japan), and other from GW Pharmaceuticals plc (Cambridge, UK) outside the submitted work. Shirley Thomas reports research grants from NIHR and The Stroke Association outside the submitted work. Ineke van der Meulen reports grants from Stichting Rotterdams Kinderrevalidatiefonds Adriaanstichting and others from Stichting Afasie Nederland, Stichting Coolsingel, and Bohn Stafleu van Loghum during the conduct of the study. Linda Worrall reports a grant from the National Health and Medical Research Council of Australia. All other authors declare no competing interests.

#### Role of the funders

The RELEASE funders had no role in the study design, data collection, analysis or interpretation, reporting, or publication processes. The methodological decision making, and analysis data were shared with co-authors. The corresponding author had final responsibility for the decision to submit for publication. The views and opinions expressed herein are those of the authors and do not necessarily reflect those of the NIHR, NHS, or the Department of Health, UK or the CSO and the Department of Health and Social Care, Scotland. All members of the RELEASE collaboration had the opportunity to review and critically appraise the final draft of the report.

#### Data Availability

To ensure adherence to primary and meta-dataset ethical approvals and minimize the risk of unintentionally sharing information that can be used to re-identify personal information, a subset of the data utilized in this study is available via the Collaboration of Aphasia Trialists [www.aphasiatrials.org](http://www.aphasiatrials.org/).

### Supplementary Materials P

*Acknowledgements*

- We acknowledge the time and effort of people aphasia to inform the primary dataset activities and whose data has in turn informed this IPD meta-analysis.
- Our IPD meta-analysis builds on the efforts of the contributing primary researchers and their generosity and collaborative approach to data sharing for the benefit of people with aphasia, their families and healthcare professionals.
- The Collaboration of Aphasia Trialists (IS1208) EU Cooperation in Science and Technology and The Tavistock Trust for Aphasia provided important infrastructural support to develop and conduct this research in addition to the funding support to conduct the research (reported in the paper).
- We thank the members of the **Aphasia Research Collaboration** Patient and Public Involvement, Norwich, group for their review of the proposed project, database creation, data extraction and analysis plans, the study findings and dissemination plans.
- Jaclyn MacArthur for administrative support in preparation of the manuscript.
